# Kindlin-2 promotes Src-mediated tyrosine phosphorylation of androgen receptor and contributes to breast cancer progression

**DOI:** 10.1038/s41419-022-04945-z

**Published:** 2022-05-20

**Authors:** Luyao Ma, Yeteng Tian, Tao Qian, Wenjun Li, Chengmin Liu, Bizhu Chu, Qian Kong, Renwei Cai, Panzhu Bai, Lisha Ma, Yi Deng, Ruijun Tian, Chuanyue Wu, Ying Sun

**Affiliations:** 1 Department of Biology, School of Life Sciences, Guangdong Provincial Key Laboratory of Cell Microenvironment and Disease Research, Shenzhen Key Laboratory of Cell Microenvironment, Southern University of Science and Technology, Shenzhen, 518055 China; 2 Department of Chemistry, Southern University of Science and Technology, Shenzhen, 518055 China; 3 Department of Pathology, School of Medicine and University of Pittsburgh Cancer Institute, University of Pittsburgh, Pittsburgh, PA 15260 USA

**Keywords:** Cell signalling, Breast cancer

## Abstract

Androgen receptor (AR) signaling plays important roles in breast cancer progression. We show here that Kindlin-2, a focal adhesion protein, is critically involved in the promotion of AR signaling and breast cancer progression. Kindlin-2 physically associates with AR and Src through its two neighboring domains, namely F1 and F0 domains, resulting in formation of a Kindlin-2-AR-Src supramolecular complex and consequently facilitating Src-mediated AR Tyr-534 phosphorylation and signaling. Depletion of Kindlin-2 was sufficient to suppress Src-mediated AR Tyr-534 phosphorylation and signaling, resulting in diminished breast cancer cell proliferation and migration. Re-expression of wild-type Kindlin-2, but not AR-binding-defective or Src-binding-defective mutant forms of Kindlin-2, in Kindlin-2-deficient cells restored AR Tyr-534 phosphorylation, signaling, breast cancer cell proliferation and migration. Furthermore, re-introduction of phosphor-mimic mutant AR-Y534D, but not wild-type AR reversed Kindlin-2 deficiency-induced inhibition of AR signaling and breast cancer progression. Finally, using a genetic knockout strategy, we show that ablation of Kindlin-2 from mammary tumors in mouse significantly reduced AR Tyr-534 phosphorylation, breast tumor progression and metastasis in vivo. Our results suggest a critical role of Kindlin-2 in promoting breast cancer progression and shed light on the molecular mechanism through which it functions in this process.

## Introduction

Breast cancer is one of the most frequently diagnosed cancers worldwide with more than 2.26 million newly diagnosed cases and over 680,000 death cases in 2020 [1]. A large number of genetic mutations and alterations of multiple signaling pathways have been found to contribute to the development and progression of breast cancer [2–8]. Among them, activation of AR has been increasingly recognized as a key event in breast cancer progression [9–12]. AR can be activated by either ligand-dependent or ligand-independent mechanism [9, 13–17]. Binding of androgen to AR leads to modulation of a series of post-receptor changes including ion transportation, membrane flexibility and signal transduction [15, 18, 19]. AR can also be activated by non-receptor tyrosine kinase-dependent pathway when the level of the ligands is low or in the absence of ligands [16, 19]. It has been well documented, for example, Tyr-534 phosphorylation of AR by Src, an oncogenic tyrosine kinase, is critical for AR activation, nuclear translocation, transactivation of downstream target genes and consequently cancer progression [13, 14, 16, 19, 20]. There is growing evidence indicating crucial roles of Src in tumor malignancy and patients’ poor outcome [21–26]. Indeed, Src and its regulatory pathways have been proposed and extensively studied as targets for therapeutic control of cancer progression [25–35]. However, despite its importance in cancer biology and therapy, how Src-mediated Tyr-534 phosphorylation of AR is regulated remains incompletely understood.

We previously showed that Kindlin-2, a key component of cell-extracellular matrix adhesions [36–47], physically interacts with Src [48]. Consistent with this, the expression of Kindlin-2, like that of Src, is frequently increased in different types of cancers including prostate, gastric, lung and breast cancer [20, 49–62]. Structurally, Kindlin-2 contains a FERM domain that is composed of four subdomains, namely F0, F1, F2 and F3 [36, 40, 48, 63–67]. We have shown that the binding of Src is mediated by the N-terminal-most F0 subdomain of Kindlin-2 [36]. The elevated expression of Kindlin-2 in cancers and its interaction with Src raise an interesting possibility that Kindlin-2 may coordinate with Src in regulation of protein tyrosine phosphorylation and promotion of cancer progression.

In this study, we have employed a photo-pTyr-scaffold strategy to investigate the role of Kindlin-2 in protein tyrosine phosphorylation and identified AR as a key target of Kindlin-2-mediated regulation of protein tyrosine phosphorylation. Furthermore, we provide evidence showing that Kindlin-2 forms a supramolecular complex with both AR and Src and thereby promotes Src-mediated Tyr-534 phosphorylation of AR, breast cancer cell migration and proliferation. Finally, using a genetic knockout strategy, we show that Kindlin-2 is crucial for AR signaling and breast cancer progression in vivo. Our studies provide a rationale for targeting the Kindlin-2-Src-AR signaling axis in therapeutic control of breast cancer progression.

## Results

### Kindlin-2 forms a supramolecular complex with AR and Src

**Fig. 1:**
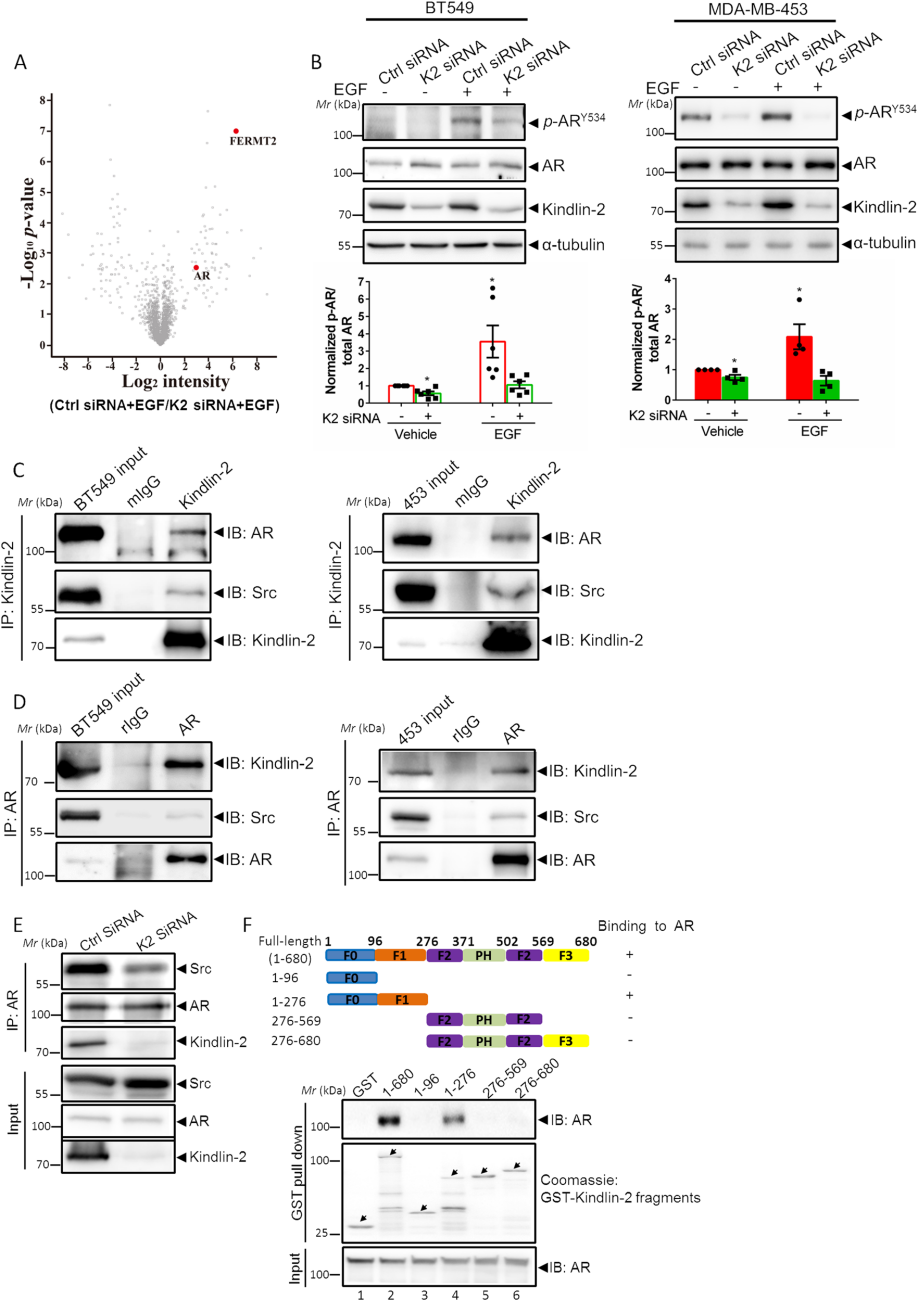
Kindlin-2 acts as a scaffold to promote the AR-Src association and facilities Src-mediated AR Tyr-534 phosphorylation. **A** Volcano plots of the enriched native tyrosine phosphorylation proteins upon EGF stimulation in BT549 cells transfected with control siRNA (Ctrl siRNA) or Kindlin-2 siRNA (K2 siRNA) using Src superbinder Photo-pTyr-scaffold approach [86]. Red dots indicated the significantly enriched AR and Kindlin-2 (FERMT2). A complete list of the enriched proteins in control vs. Kindlin-2 knockdown BT549 cells was shown in Supplementary Table S1. False discovery rate (FDR) = 0.05, s0 = 2, two-sample *t*-test, *n* = 2. **B** Immunoblotting analysis of total AR and AR Tyr-534 phosphorylation level in control (Ctrl siRNA) and Kindlin-2 knockdown (K2 siRNA) BT549 cells or MDA-MB-453 cells with or without EGF stimulation (upper panel). Quantification data were shown in the lower panel. *p < 0.05 vs. Ctrl siRNA, *n* = 6 for BT549 cells; *n* = 4 for MDA-MB-453 cells. **C** BT549 (left panel) or MDA-MB-453 (right panel) cell lysates were immunoprecipitated with anti-Kindlin-2 antibody or mouse control IgG (mIgG) followed by immunoblotting with antibodies as indicated. The presence of Kindlin-2, AR and Src in cell lysates was shown as input. **D** BT549 (left panel) or MDA-MB-453 (right panel) cell lysates were immunoprecipitated with anti-AR antibody or rabbit control IgG (rIgG) followed by immunoblotting with antibodies as indicated. The presence of Kindlin-2, AR and Src in cell lysates was shown as input. **E** Control (Ctrl siRNA) and Kindlin-2 knockdown (K2 siRNA) BT549 cells lysates were immunoprecipitated with anti-AR antibody followed by immunoblotting with antibodies as indicated. The presence of Kindlin-2, AR and Src in cell lysates was shown as input. **F** Mapping the subdomains of Kindlin-2 that mediated the association with AR. Upper panel: schematic illustration of various Kindlin-2 fragments that were used in the GST pull-down assay. Lower panel: GST-fusion proteins containing various fragments of Kindlin-2 were used to pull-down endogenous AR from BT549 cell lysates. K2 Kindlin-2, AR androgen receptor.

Phosphotyrosine(pTyr)-regulated protein complexes are key factors for regulating signaling transduction during cancer progression [68–70]. To investigate the function of Kindlin-2 in the regulation of pTyr signaling in breast cancer, we applied the photo-pTyr-scaffold approach to capture Kindlin-2-regulated native pTyr protein complexes in breast cancer cells (see “Material and Methods” section for detail). To do this, we treated wild-type or Kindlin-2 knockdown breast cancer cell BT549 with EGF for 5 min to activate EGF-induced protein tyrosine phosphorylation and then applied the Src superbinder (engineered Src kinase SH_2_ domain) photo-pTyr-scaffold to capture Kindlin-2 regulated pTyr protein complexes in response to EGF stimulation by mass spectrometry (MS). A number of pTyr proteins were captured by the Src superbinder photo-pTyr-scaffold efficiently in wild-type BT549 cells but much less efficiently in Kindlin-2 knockdown BT549 cells (Fig. 1A and Supplementary Table S1), suggesting a direct or indirect role of Kindlin-2 in regulation of EGF-induced tyrosine phosphorylation of these proteins in breast cancer cells. These pTyr proteins participate in a variety of cellular signaling events including cell adhesion (e.g., integrin β4), cytoskeletal organization (e.g., filamin-C), protein synthesis (e.g., elongation factor 1-alpha 2), metabolism (e.g., NADH dehydrogenase) and signaling (e.g., AR) (Supplementary Table S1). Given the crucial role of AR in breast cancer progression [9–12] and the fact that AR is a substrate of Src [13, 14, 16, 19, 20], to which Kindlin-2 binds [48], we decided to focus on the role and mechanism of Kindlin-2-mediated regulation of AR tyrosine phosphorylation, signaling and breast cancer progression in the current study. EGF is known to induce two prominent and functionally important tyrosine phosphorylation sites on AR, one is located at Y267 that is phosphorylated by Ack kinase and the other is located at Y534 that is phosphorylated by Src kinase [13, 16, 71, 72]. To examine which site of tyrosine phosphorylation was regulated by Kindlin-2, we analyzed the effect of Kindlin-2 on Tyr-267 and Try-534 phosphorylation of AR in two AR-positive human breast cancer cell lines, BT549 and MDA-MB-453, respectively. Immunoblotting analysis showed that knockdown of Kindlin-2 did not significantly alter AR total protein level (Fig. 1B). However, the level of AR Tyr-534 phosphorylation (Fig. 1B), but not that of AR Tyr-267 phosphorylation (Supplementary Fig. 1), was remarkably reduced in Kindlin-2 knockdown cells compared with BT549 or MDA-MB-453 control cells, especially in the presence of EGF stimulation, suggesting that Kindlin-2 is critical for regulation of AR Tyr-534 phosphorylation. Because AR Tyr-534 is known to be phosphorylated by Src kinase in response to EGF treatment [13, 16] and Kindlin-2 is known to interact with Src [48], we hypothesized that Kindlin-2 might serve as a scaffold protein to facilitate the formation of a supramolecular complex containing Kindlin-2, Src and AR in cells and thereby promote Src-mediated AR Tyr-534 phosphorylation. To test this, we carried out sequential immunoprecipitation (IP) experiments with either anti-Kindlin-2 or anti-AR antibodies in breast cancer cells. The results showed that both endogenous AR and Src were readily co-IPed with Kindlin-2 in both BT549 and MDA-MB-453 cells (Fig. 1C). Reciprocally, endogenous Kindlin-2 and Src were co-IPed with AR (Fig. 1D). These results suggest that Kindlin-2, AR and Src indeed form a supramolecular complex. To further test this, we depleted Kindlin-2 from breast cancer cells and analyzed the effect on the association of Src with AR. The results showed that depletion of Kindlin-2 significantly reduced the complex formation between Src and AR (Fig. 1E), suggesting that Kindlin-2 is critical for this process.

Next, we sought to identify the Kindlin-2 subdomain that is critical for the complex formation with AR. To do this, we prepared GST-fusion proteins containing various subdomains of Kindlin-2 and tested their abilities to associate with AR. The results showed that both the full-length Kindlin-2 and the F0F1 subdomains (Fig. 1F, lanes 2 and 4), but neither the F0 nor the F2F3 subdomains (Fig. 1F, lanes 3 and 6), pulled down AR, suggesting that F1 subdomain, but not F0, F2 or F3, is critical for Kindlin-2 association with AR.

### Kindlin-2 regulates AR downstream signaling

**Fig. 2:**
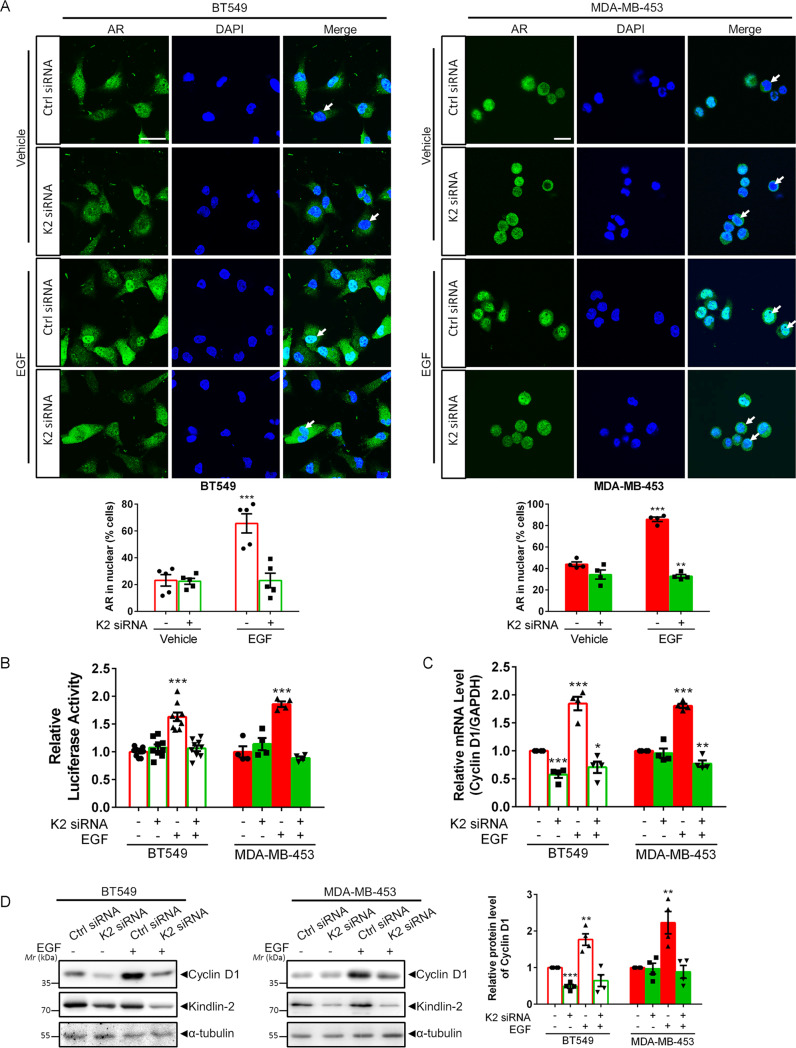

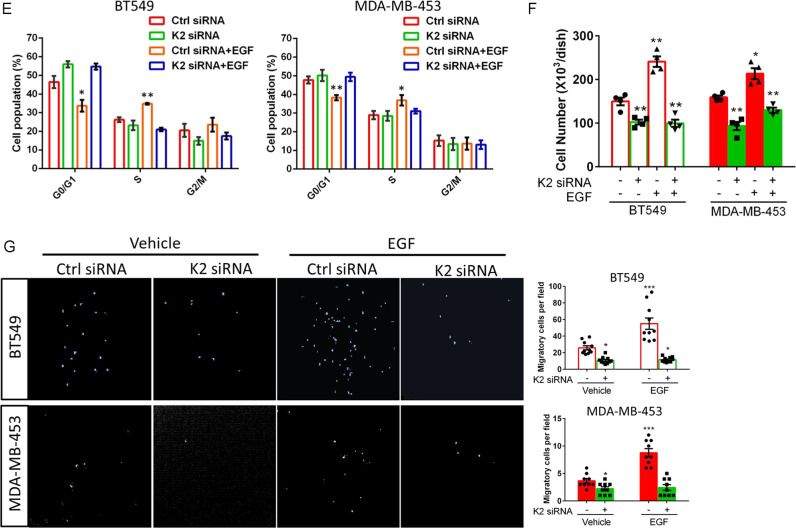
Loss of Kindlin-2 inhibits AR transcriptional activity and its downstream events. **A** Representative immunofluorescence staining of AR (green) in control (Ctrl siRNA) and Kindlin-2 knockdown (K2 siRNA) BT549 cells (left panel) or MDA-MB-453 cells (right panel) with or without EGF stimulation. Cell nuclei were visualized with DAPI (blue). Arrows mark the nuclear regions. Scale bar: 50 µm. Quantification analysis of the percentage of cells with AR nuclear staining was shown in the lower panel. **p < 0.01, ***p < 0.001 vs. Ctrl siRNA, *n* = 5 independent experiments for BT549 cells; *n* = 4 independent experiments for MDA-MB-453 cells. **B** Luciferase analysis of AR transcriptional activity in control (Ctrl siRNA) and Kindlin-2 knockdown (K2 siRNA) BT549 cells or MDA-MB-453 cells with or without EGF stimulation. Relative luminance was calculated by luminance signal relative to total protein concentration. ***p < 0.001 vs. Ctrl siRNA, *n* = 9 for BT549 cells; *n* = 4 for MDA-MB-453 cells. **C** qPCR analysis of *cyclin D1* mRNA expression in control (Ctrl siRNA) and Kindlin-2 knockdown (K2 siRNA) BT549 cells or MDA-MB-453 cells with or without EGF stimulation. *p < 0.05, **p < 0.01, ***p < 0.001 vs. Ctrl siRNA, *n* = 4. **D** Immunoblotting analysis of cyclin D1 protein expressio*n* in control (Ctrl siRNA) and Kindlin-2 knockdown (K2 siRNA) BT549 cells or MDA-MB-453 cells with or without EGF stimulation (left panel). Quantification of cyclin D1 protein expression was shown at the right panel. **p < 0.01, ***p < 0.001 vs. Ctrl siRNA, *n* = 4. **E** Quantification analysis of cell cycle transition in control (Ctrl siRNA) and Kindlin-2 knockdown (K2 siRNA) BT549 cells (left panel) or MDA-MB-453 cells (right panel) with or without EGF stimulation. Cells with 2n signal were in G0/G1 phases; with 4n signal were in G2/M phases; between 2n to 4n signal were in S phase. *p < 0.05, **p < 0.01 vs. Ctrl siRNA, *n* = 5. **F** Quantification analysis of cell proliferation assay in the indicated cells, as described in “Materials and Methods”. *p < 0.05, **p < 0.01 vs. Ctrl siRNA, *n* = 4. **G** Cell migration was measured by transwell cell migration assay, as described in “Materials and Methods”. Representative images (left panel) and quantification analysis (right panel) were shown. *p < 0.05, ***p < 0.001 vs. Ctrl siRNA, *n* = 10 for BT549 cells; *n* = 9 for MDA-MB-453 cells. Original magnification, ×100. K2 Kindlin-2, AR androgen receptor, DAPI 4,6-diamidino-2-phenylindole.

We further investigated the functional significance of the complex formation between Kindlin-2, Src and AR in AR signaling. Src-mediated AR Tyr-534 phosphorylation is known to induce AR nuclear translocation, active its transcriptional activity, and drive cancer cell growth in response to EGF stimulation [13]. Because depletion of Kindlin-2 impairs AR association with Src (Fig. 1E) and AR Tyr-534 phosphorylation (Fig. 1B), we reasoned that Kindlin-2 deficiency might also influence AR nuclear translocation and tested this experimentally. As shown in Fig. 2A, EGF treatment promoted AR nuclear translocation in both BT549 and MDA-MB-453 wild-type cells, whereas the majority of AR remained in the cytoplasm in Kindlin-2 knockdown cells despite the presence of EGF. Because AR nuclear translocation is crucial for AR transcriptional activity, we also analyzed the effect of Kindlin-2 on EGF-induced AR transcriptional activity. The results showed that EGF increased AR transcriptional activity by ~1.6-fold in BT549 cells and 1.8 fold in MDA-MB-453 wild-type cells (Fig. 2B), which was completely blocked in Kindlin-2 knockdown cells, suggesting that Kindlin-2 is indeed critical for EGF-induced AR signaling. To further test this, we assessed the effects of Kindlin-2 knockdown on the transcription of *cyclin D1*, a well-known AR target gene that promotes breast cancer progression and metastasis [17, 73–77]. The results showed that the mRNA level of *cyclin D1* was significantly reduced in response to knockdown of Kindlin-2 upon EGF stimulation (Fig. 2C). Consistent with the down-regulation of the mRNA level, the protein level of cyclin D1 was also diminished in Kindlin-2 knockdown cells compared with that in both BT549 and MDA-MB-453 wild-type cells under the EGF treated conditions (Fig. 2D). Because of the crucial role of cyclin D1 in regulating cell cycle transition from G1 to S phase, cell proliferation and migration, we performed cell cycle assay to test experimentally whether cell cycle transition was affected by the deficiency of Kindlin-2. The results showed that knockdown of Kindlin-2 in both BT549 and MDA-MB-453 stunted EGF-induced breast cancer cell cycle progression (Fig. 2E). Consistent with the effect on the cell cycle progression, Kindlin-2 knockdown in both BT549 and MDA-MB-453 cells significantly inhibited breast cancer cell growth (Fig. 2F). Furthermore, breast cancer cell migration was also inhibited in response to Kindlin-2 knockdown (Fig. 2G). Collectively, these results suggest that Kindlin-2 is critical for regulation of AR signaling, breast cancer cell proliferation and migration.

### Kindlin-2 association with AR is crucial for AR Tyr-534 phosphorylation and AR signaling

**Fig. 3:**
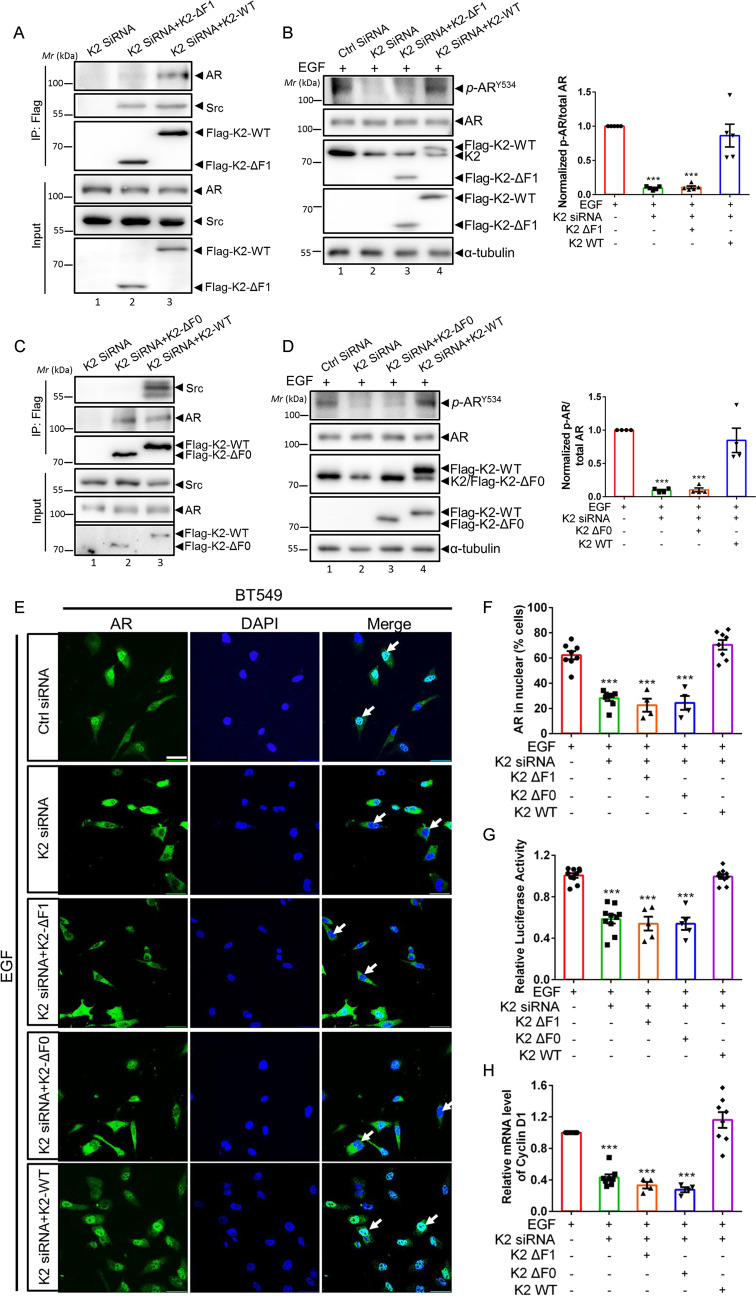

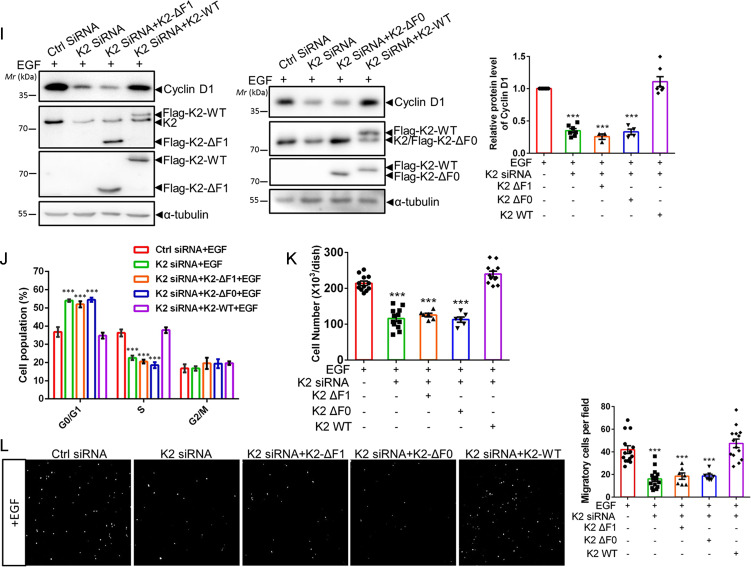
Kindlin-2-ΔF1 and Kindlin-2-ΔF0 mutants fail to rescue AR Tyr-534 phosphorylation and downstream signaling. Control (Ctrl siRNA) or Kindlin-2 knockdown (K2 siRNA) BT549 cells was infected with lentiviral vectors encoding wild-type Kindlin-2 (K2-WT), F1-domain-deleted mutant of Kindlin-2 (K2-ΔF1), F0-domain-deleted mutant of Kindlin-2 (K2-ΔF0) or empty vector 3×Flag-tagged pLVX-IRES-Hyg. **A** Cell lysates were immunoprecipitated with anti-Flag antibody followed by immunoblotting with antibodies as indicated. The presence of Flag-tagged-wild-type Kindlin-2 (Flag-K2-WT), Flag-tagged-F1-domain-deleted mutant of Kindlin-2 (Flag-K2-ΔF1), AR and Src in cell lysates was shown as input. **B** Immunoblotting analysis of AR Tyr-534 phosphorylation level in cells as specified in the figure (left panel). Quantification analysis of the normalized ratio of AR Tyr-534 phosphorylation level to total AR was shown in the right panel. ***p < 0.001 vs. Ctrl siRNA + EGF, *n* = 5. **C** Cell lysates were immunoprecipitated with anti-Flag antibody followed by immunoblotting with antibodies as indicated. The presence of Flag-K2-WT, Flag-tagged-F0-domain-deleted mutant of Kindlin-2 (Flag-K2-ΔF0), AR and Src in cell lysates was shown as input. **D** Immunoblotting analysis of AR Tyr-534 phosphorylation level in cells as specified in the figure (left panel). Quantification analysis of the normalized ratio of AR Tyr-534 phosphorylation level to total AR was shown in the right panel. ***p < 0.001 vs. Ctrl siRNA + EGF, *n* = 4. **E** Representative immunofluorescence staining of AR in cells (as specified in the figure). Arrows mark the nuclear regions. Scale bar: 50 µm. Cell nuclei were visualized with DAPI (blue). **F** Quantification analysis of the percentage of cells (as specified in the figure) with AR nuclear staining was shown. ***p < 0.001 vs. Ctrl siRNA, *n* = 8 for Ctrl siRNA + EGF, K2 siRNA *+* EGF and K2 siRNA + K2-WT + EGF; *n* = 4 for K2 siRNA + K2-ΔF1 + EGF and K2 siRNA + K2-ΔF0 + EGF. **G** Luciferase analysis of AR transcriptional activity in cells (as specified in the figure). Relative luminance was calculated by luminance signal relative to total protein concentration. ***p < 0.001 vs. Ctrl siRNA + EGF, *n* = 10 for Ctrl siRNA + EGF, K2 siRNA + EGF and K2 siRNA + K2-WT + EGF; *n* = 5 for K2 siRNA + K2-ΔF1 + EGF and K2 siRNA + K2*-*ΔF0 + EGF. **H** qPCR analysis of *cyclin D1* mRNA expression. ***p < 0.001 vs. Ctrl siRNA + EGF, *n* = 8 for Ctrl siRNA + EGF, K2 siRNA + EGF and K2 siRNA+ K2*-*WT + EGF; *n* = 4 for K2 siRNA + K2-ΔF1 + EGF and K2 siRNA + K2-ΔF0 + EGF. **I** Immunoblotting analysis of cyclin D1 protein expression in cells (as specified in the figure) (left panel). Quantification of cyclin D1 protein expression was shown at the right panel. ***p < 0.001 vs. Ctrl siRNA + EGF, *n* = 7 for Ctrl siRNA + EGF, K2 siRNA + EGF and K2 siRNA + K2-WT + EGF; *n* = 3 for K2 siRNA + K2-ΔF1 + EGF; *n* = 4 for K2 siRNA + K2-ΔF0 + EGF. **J** Quantification analysis of cell cycle transition in cells (as specified in the figure). Cells with 2n signal were in G0/G1 phases; with 4n signal were in G2/M phases; between 2n to 4n signal were in S phase. ***p < 0.001 vs. Ctrl siRNA + EGF, *n* = 8 for Ctrl siRNA + EGF, K2 siRNA + EGF and K2 siRNA + K2-WT + EGF; *n* = 4 for K2 siRNA + K2-ΔF1 + EGF and K2 siRNA + K2-ΔF0 + EGF. **K** Quantification analysis of cell proliferation assay in the indicated cells, as described in “Materials and Methods”. ***p < 0.001 vs. Ctrl siRNA + EGF, *n* = 12 for Ctrl siRNA + EGF, K2 siRNA + EGF and K2 siRNA+ K2-WT + EGF; *n* = 6 for K2 siRNA + K2-ΔF1 + EGF and K2 siRNA + K2-ΔF0 + EGF. **L** Cell migration was measured by transwell cell migration assay, as described in “Materials and Methods”. Representative images (left panel) and quantification analysis (right panel) were shown. ***p < 0.001 vs. Ctrl siRNA + EGF, *n* = 14 for Ctrl siRNA + EGF, K2 siRNA + EGF and K2 siRNA+ K2-WT + EGF; *n* = 7 for K2 siRNA + K2-ΔF1 + EGF and K2 siRNA + K2-ΔF0 + EGF. Original magnification, ×100. K2 Kindlin-2, AR androgen receptor.

We next tested whether Kindlin-2-AR association is involved in the regulation of AR Tyr-534 phosphorylation and downstream signaling. To do this, we expressed either full-length Kindin-2 or a Kindlin-2 mutant in which the F1 subdomain was deleted (referred to as Kindlin-2-ΔF1 hereafter) in Kindlin-2 knockdown BT549 cells (Fig. 3A, lanes 2 and 3). Consistent with the results from the GST-fusion protein pull-down experiments (Fig. 1F), co-IP experiments showed that Kindlin-2-ΔF1 mutant, unlike that of the full-length Kindlin-2, failed to associate with AR (Fig. 3A, lane 2). In contrast, both full-length Kindin-2 and Kindlin-2-ΔF1 mutant associated with Src (Fig. 3A, lanes 2 and 3). These results suggest that the F1 subdomain is crucial for the association of Kindlin-2 with AR but not that with Src. Functionally, whilst overexpression of full-length Kindlin-2 in Kindlin-2 knockdown cells successfully reversed the inhibition of EGF-induced AR Tyr-534 phosphorylation, overexpression of Kindlin-2-ΔF1 failed to do so (Fig. 3B, lanes 3 and 4), suggesting that the F1 subdomain is critical for AR Tyr-534 phosphorylation. Consistently, re-expression of the AR association defective Kindlin-2-ΔF1, unlike that of full-length Kindlin-2, in Kindlin-2 deficient cells failed to restore the defect of AR nuclear translocation (Fig. 3E, F). In line with this, Kindlin-2 deficiency-induced inhibition of AR transcriptional activity was reversed by the overexpression of full-length Kindlin-2 but not that of Kindlin-2-ΔF1 mutant (Fig. 3G). Likewise, Kindlin-2 deficiency-induced inhibition on the mRNA and protein expression of cyclin D1 was also reversed by the overexpression of full-length Kindlin-2 but not that of Kindlin-2-ΔF1 mutant (Fig. 3H, I). Furthermore, Kindlin-2-ΔF1 mutant, unlike full-length Kindlin-2, failed to rescue the inhibition on G1/S phase transition, cell proliferation and migration caused by the depletion of Kindlin-2 (Fig. 3J–L). Collectively, these results suggest that Kindlin-2 association with AR is critical for Src-mediated AR Tyr-534 phosphorylation, AR nuclear translocation, AR target gene expression, breast cancer cell proliferation and migration.

### Kindlin-2 interaction with Src is crucial for AR Tyr-534 phosphorylation and AR signaling

Our previous studies have shown that the F0 subdomain of Kindlin-2 mediates the interaction between Kindlin-2 and Src [48]. To test whether Kindlin-2 interaction with Src is involved in the regulation of AR signaling, we generated F0 deletion mutant of Kindlin-2 (referred to as Kindlin-2-ΔF0 hereafter). We first analyzed the ability of Kindlin-2-ΔF0 to associate with Src and AR in BT549 cells by co-immunoprecipitation experiments. As expected, deletion of F0 completely abolished the association of Kindlin-2 with Src (Fig. 3C, lane 2). In contrast, Kindlin-2-ΔF0 mutant, like full-length Kindlin-2, was able to associate with AR (Fig. 3C, lanes 2 and 3), indicating that the F0 subdomain is required for the association of Kindlin-2 with Src but not that with AR. Next, we investigated whether expression of Kindlin-2-ΔF0 in Kindlin-2 knockdown cells was able to restore AR tyrosine phosphorylation and promote its downstream signaling events. Figure 3D showed that expression of Kindlin-2-ΔF0 mutant (Fig. 3D, lane 3), unlike that of wild-type Kindlin-2 (Fig. 3D, lane 4), was unable to reverse the inhibition of EGF-induced AR Tyr-534 phosphorylation caused by loss of Kindlin-2. Furthermore, expression of Kindlin-2-ΔF0, unlike that of wild-type Kindlin-2, failed to reverse Kindlin-2-deficiency-induced defects in AR nuclear translocation (Fig. 3E, F), AR transcriptional activity (Fig. 3G), cyclin D1 expression (Fig. 3H, I), G1/S phase transition (Fig. 3J), breast cell proliferation (Fig. 3K) and migration (Fig. 3L). Collectively, these results suggest that the association of Kindlin-2 with Src, like that with AR, is critical for regulation of AR signaling, breast cancer cell proliferation and migration.

### AR Tyr-534 phosphorylation is critical for Kindlin-2-mediated regulation of breast cancer progression

**Fig. 4:**
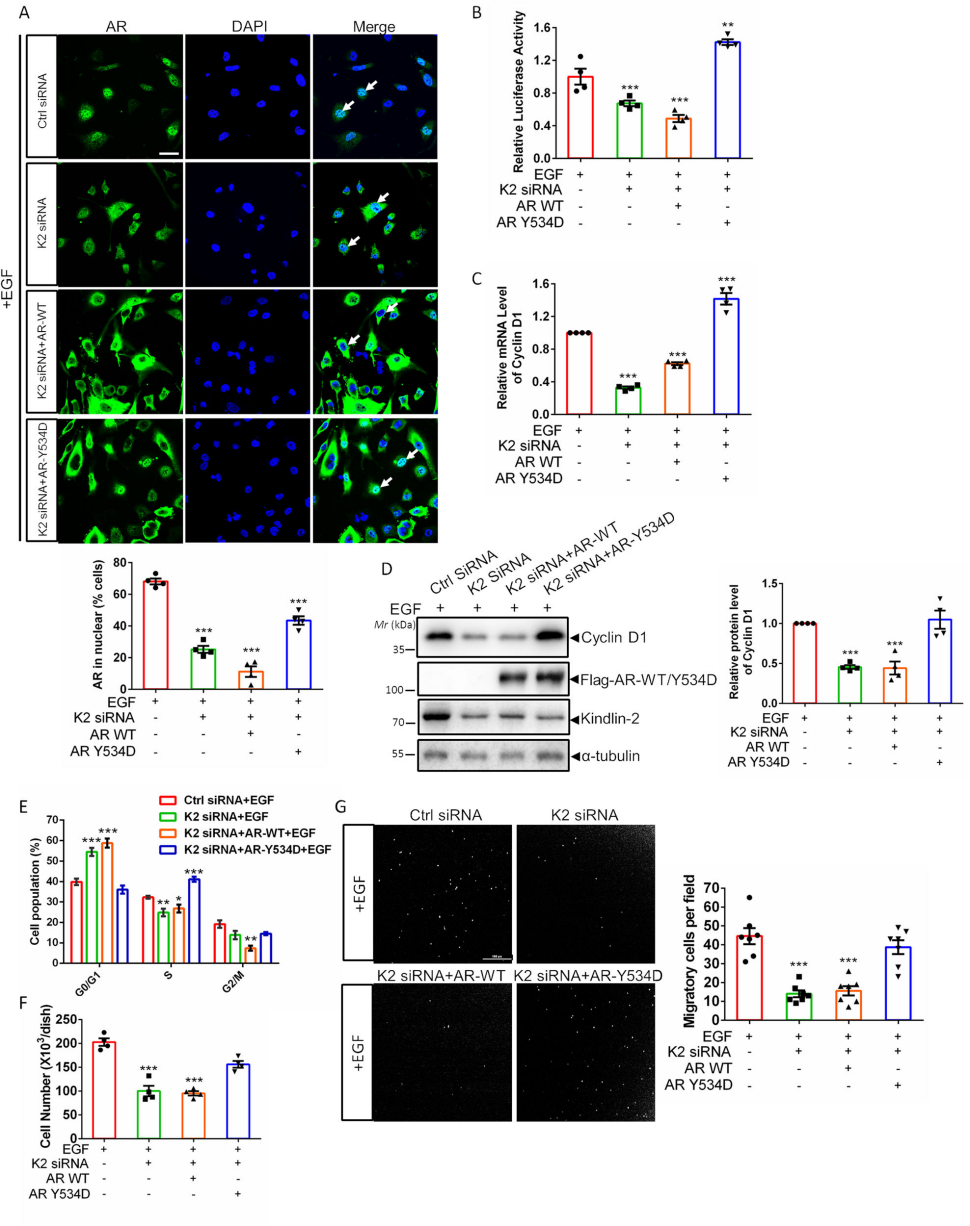
AR-Y534D mutant but not wild-type AR rescues Kindlin-2 deficiency-induced inhibition on AR signaling and breast cancer progression. Control (Ctrl siRNA) or Kindlin-2 knockdown (K2 siRNA) BT549 cells was infected with lentiviral vectors encoding wild-type AR (AR-WT), phosphor-mimic mutant of AR (AR-Y534D) or empty vector 3×Flag-tagged pLVX-IRES-Hyg. **A** Representative immunofluorescence staining of AR in cells (as specified in the figure). Arrows mark the nuclear regions. Scale bar: 50 µm. Cell nuclei were visualized with DAPI (blue). Quantification analysis of the percentage of cells with AR nuclear staining was shown in the lower panel. ***p < 0.001 vs. Ctrl siRNA, *n* = 4 indepe*n*dent experiments. **B** Luciferase analysis of AR transcriptional activity in cells (as specified in the figure). Relative luminance was calculated by luminance signal relative to total protein concentration. **p < 0.01, ***p < 0.001 vs. Ctrl siRNA + EGF, *n* = 4. **C** qPCR analysis of *cyclin D1* mRNA expression. ***p < 0.001 vs. Ctrl siRNA + EGF, *n* = 4. **D** Immunoblotting analysis of cyclin D1 protein expression in cells (as specified in the figure) (left panel). Quantification of cyclin D1 protein expression was shown at the right panel. ***p < 0.001 vs. Ctrl siRNA + EGF, *n* = 4. **E** Quantification analysis of cell cycle transition in cells (as specified in the figure). Cells with 2n signal were in G0/G1 phases; with 4n signal were in G2/M phases; between 2n to 4n signal were in S phase. *p < 0.05, **p < 0.01, ***p < 0.001 vs. Ctrl siRNA + EGF, *n* = 4. **F** Quantification analysis of cell proliferation assay of indicated cells. ***p < 0.001 vs. Ctrl siRNA + EGF, *n* = 4. **G** Cell migration was measured by transwell cell migration assay, as described in “Materials and Methods”. Representative images (left panel) and quantification analysis (right panel) were shown. ***p < 0.001 vs. Ctrl siRNA + EGF, *n* = 7. Original magnification, ×100. K2 Kindlin-2, AR androgen receptor.

To explore Kindlin-2 deficiency-induced inhibition of AR signaling and breast cancer progression is due to the reduction of AR Tyr-534 phosphorylation, we used a phosphor-mimic mutant AR-Y534D (tyrosine 534 replaced by an aspartic acid) to study the function of AR Tyr-534 phosphorylation. Figure 4A showed that overexpression of phosphor-mimic mutant AR-Y534D but not wild-type AR successfully restored Kindlin-2 deficiency-induced AR nuclear translocation defect (Fig. 4A). Furthermore, re-introduction of AR-Y534D rescued Kindlin-2 deficiency-induced inhibition on AR transcriptional activity (Fig. 4B), cyclin D1 expression (Fig. 4C, D), G1/S phase transition (Fig. 4E), breast cell proliferation (Fig. 4F) and migration (Fig. 4G). In contrast, wild-type AR failed to do so (Fig. 4A–G). Taken together, these data support our hypothesis that the effects of Kindlin-2 on AR signaling and breast cancer cell behaviors are mediated, at least in part, by controlling AR Tyr-534 phosphorylation level.

### Loss of Kindlin-2 delays breast cancer progression in vivo

**Fig. 5:**
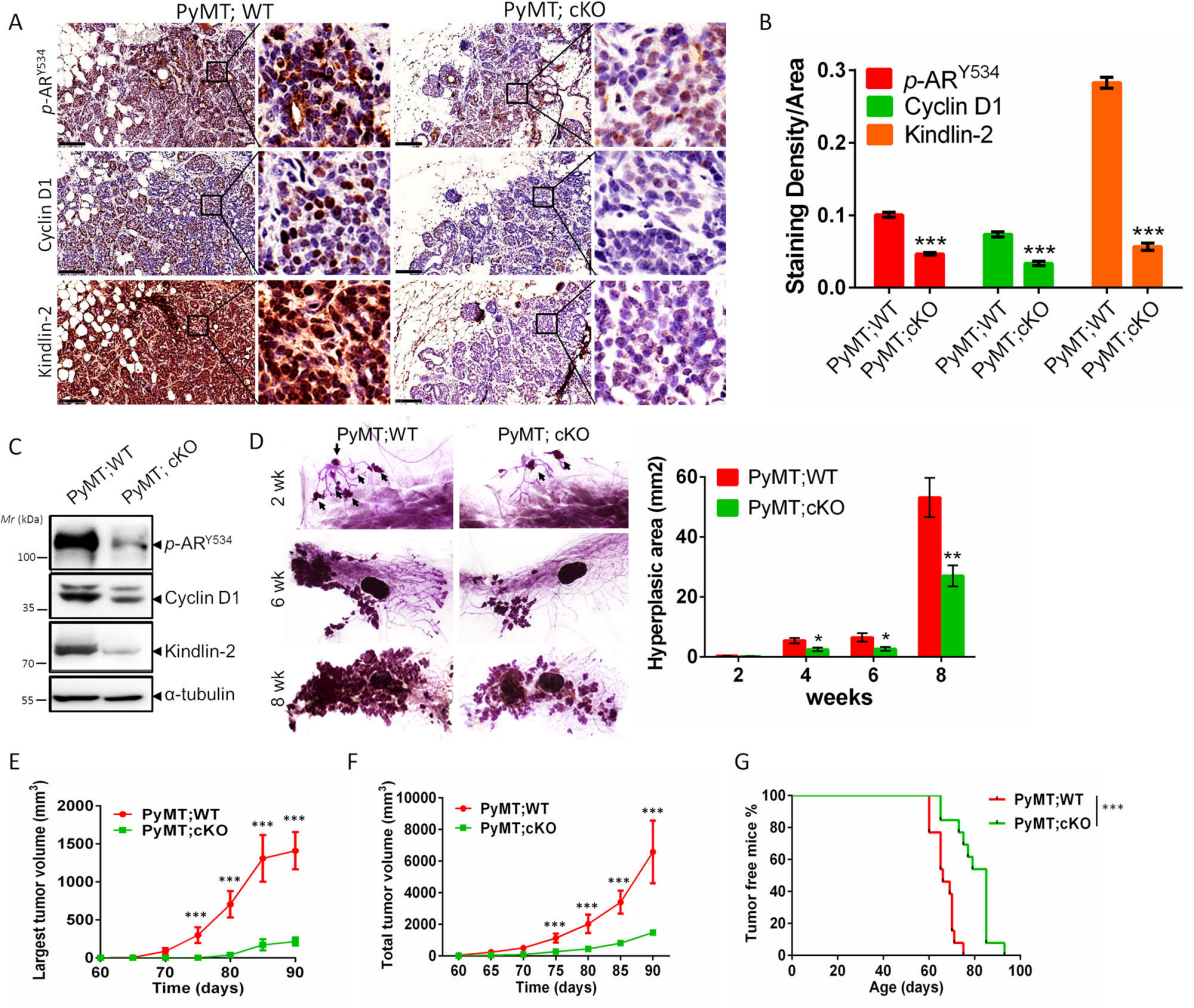
Loss of Kindlin-2 inhibits breast cancer progression in vivo. **A** Representative images of breast tumor sections from PyMT;WT and PyMT; cKO littermates at 8-week-age stained with anti-Kindlin-2, anti-cyclin D1 or anti-AR Tyr-534 phosphorylation antibodies, respectively. Scale bar: 100 µm. The right panels of **A** showed higher magnification images of the areas outlined with black squares in the left panels. **B** Quantification of staining density of Kindlin-2, cyclin D1 or AR Tyr-534 phosphorylation level in tumor sections was shown. ***p < 0.001 vs. PyMT;WT, *n* = 4 mice for PyMT; WT mice; *n* = 3 mice for PyMT; cKO mice; for each mice, the quantification was performed from at least fifteen images. **C** Immunoblotting analysis of primary tumor cells isolated from the PyMT;WT and PyMT; cKO mice with antibodies as indicated. **D** Mammary gland whole-mounts were prepared from PyMT;WT and PyMT; cKO littermates with indicated ages (left panel). Arrows indicate the hyperplastic area. Quantification of hyperplastic areas in mammary glands was shown in the right panel. *p < 0.05, **p < 0.01 vs. PyMT;WT, *n* = 8 mice at 2 weeks of age; *n* = 9 mice at 4 and 8 weeks of age*; n* = 3 mice at 6 weeks of age. Original magnification, ×30 (2 weeks); ×6.3 (6 and 8 weeks). Quantification of the largest tumor volume (**E**) and the total tumor volume (**F**) per mouse. ***p < 0.001 vs. PyMT**;**WT. *n* = 9 mice per group. **G** Kaplan–Merier tumor*-*free curves of PyMT;WT and PyMT; cKO mice. ***p < 0.001 vs. PyMT;WT, *n* = 13 mice per group. wk weeks, WT wild-type, cKO conditional knockout.

We next investigated the effects of Kindlin-2 on AR tyrosine phosphorylation and breast cancer progression in vivo. To do this, we first generated mammary epithelium-specific Kindlin-2 conditional knockout mice (referred to as Kindlin-2 cKO hereafter) using the Cre/LoxP system (MMTV-Cre), which targets exons 5 and 6 of the Kindlin-2 allele (Supplementary Fig. 2A). The Kindlin-2 cKO mice, as well as the Kindlin-2^+/+^;MMTV-Cre (referred to as WT hereafter) control mice were confirmed by PCR analysis of tail genomic DNA (Supplementary Fig. 2B). Immunohistochemistry analyses of mammary gland demonstrated that Kindlin-2 expression was markedly reduced in the mammary epithelium cells in Kindlin-2 cKO mice compared with littermate control mice (Supplementary Fig. 2C). Mice of all genotypes were born at the expected Mendelian frequency. Next, we intercrossed Kindlin-2 cKO or WT mice with MMTV-PyMT mice, a widely used breast cancer mouse model [78–81], to generate PyMT; Kindlin-2 cKO and PyMT; WT mice. Immunohistochemistry staining confirmed that Kindlin-2 was successfully ablated in the breast tumor sections from PyMT; Kindlin-2 cKO mice (Fig. 5A). Consistent with the in vitro findings (Figs. 1B and 2D), significant reduction of AR Tyr-534 phosphorylation and cyclin D1 expression was observed in the tumor sections (Fig. 5A, B) and isolated primary tumor cells (Fig. 5C) from the PyMT; Kindlin-2 cKO mice compared with those from the PyMT; WT mice, suggesting that loss of Kindlin-2 diminishes AR Tyr-534 phosphorylation and cyclin D1 expression. Because cyclin D1 expression is important for the regulation of cancer cell growth, we examine the effect of Kindlin-2 ablation on mammary tumorigenesis in vivo. As expected, the total areas of hyperplasia were reduced by ~50% in the glands of the PyMT; Kindlin-2 cKO mice compared with that in the PyMT; WT mice from 4 weeks onward (Fig. 5D). To further test the effect of Kindlin-2 ablation on breast cancer progression, tumor growth was measured every 5 days after tumor onset in the PyMT; WT and PyMT; Kindlin-2 cKO mice. Starting from 11 weeks, both the size of the largest tumor and the total tumor burden were significantly decreased in the PyMT; Kindlin-2 cKO mice compared to those in the PyMT; WT mice (Fig. 5E, F). Moreover, while 100% of the PyMT; WT mice presented palpable tumors (2-mm diameter) around day 77, the time for the PyMT; Kindlin-2 cKO mice to reach this stage was significantly delayed (to 97 days) (Fig. 5G). Collectively, these results suggest that loss of Kindlin-2 significantly delays breast cancer progression in vivo.

We have also examined the effects of Kindlin-2 deficiency on normal mammary gland development. The results showed that mammary glands from 6-, and 8-week-old WT and Kindlin-2 cKO mice presented normal ductal growth, ductal branching and morphology (Supplementary Fig. 3A, B). At pregnancy day 14, the size of lobules appeared normal both in WT and Kindlin-2 cKO mice (Supplementary Fig. 3C). These results suggest that the effects of Kindlin-2 deficiency on mammary tumor progression are not caused by alteration of normal mammary gland development.

**Fig. 6:**
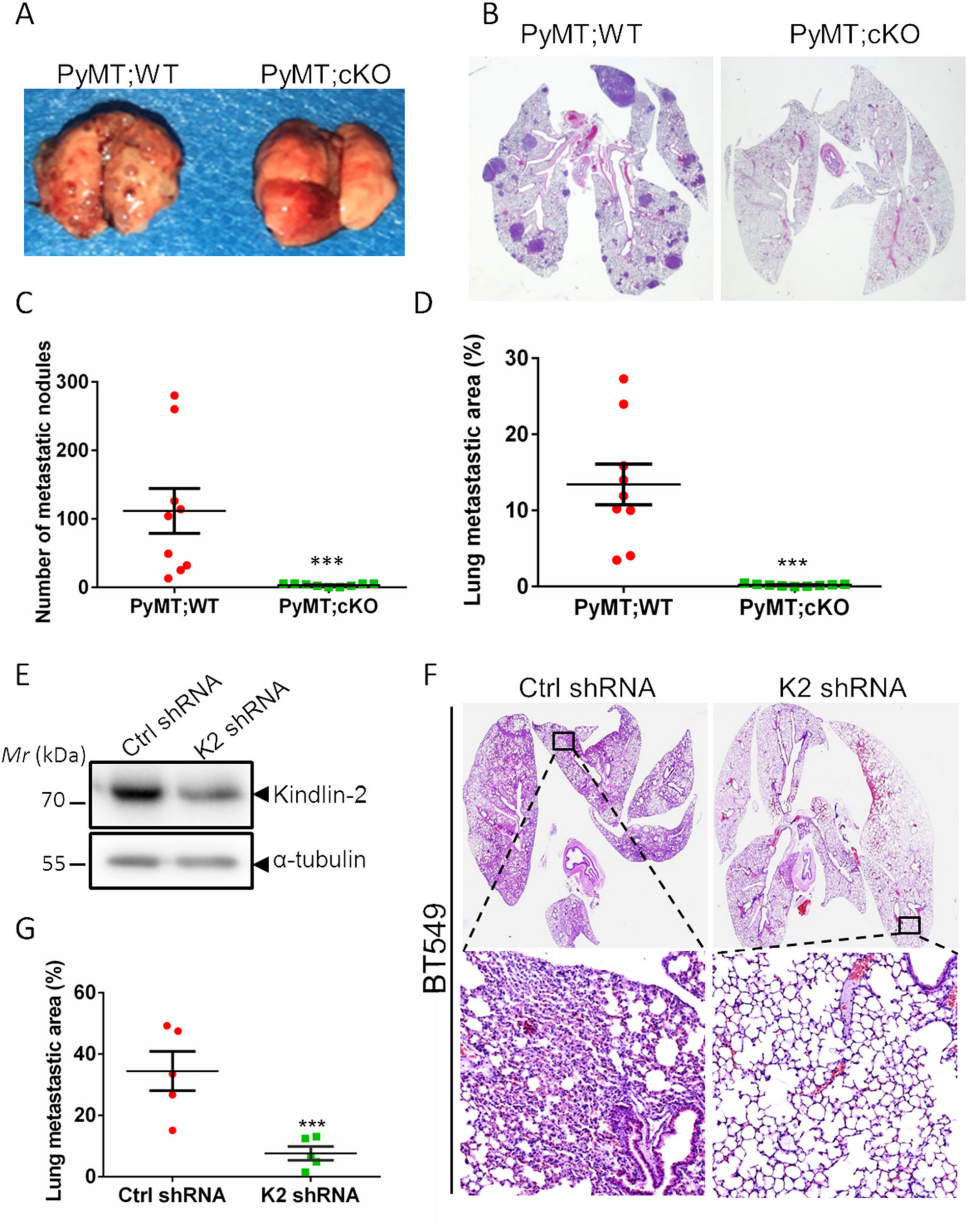
Loss of Kindlin-2 inhibits breast tumor metastasis in vivo. **A** Representative gross images of lung metastatic nodules from the PyMT;WT and PyMT; Kindlin-2 cKO littermates at 15 weeks of age. **B** Representative histological analyses of lung metastases by H&E staining. Original magnification: ×10. Quantification analysis of the number (**C**) and the areas (**D**) of lung metastatic nodules from PyMT;WT and PyMT; Kindlin-2 cKO mice at 15 weeks of age. ***p < 0.001 vs. PyMT;WT, *n* = 9. **E** BT549 cells were infected with lentiviral vectors encoding control shRNA (Ctrl shRNA) or Kindlin-2 shRNA (K2 shRNA). Five days after infection, the cells were harvested and analyzed by immunoblotting with antibodies as indicated. **F** Histological analyses of lung metastases from mice tail injection with Kindlin-2 knockdown (K2 shRNA) or control (Ctrl shRNA) BT549 cells. Original magnification: ×10 for the upper panels and ×100 for the lower panels. **G** Quantification analysis of the areas of lung metastatic nodules. ***p < 0.001 vs. Ctrl shRNA, *n* = 5.

Our in vitro studies showed that Kindlin-2 knockdown inhibits not only the proliferation but also the migration of breast cancer cells (Fig. 2G), suggesting that Kindlin-2 may function not only in the growth but also metastasis of breast tumor. To test this experimentally in vivo, we analyzed the effect of Kindlin-2 on lung metastasis using the PyMT; WT and PyMT; Kindlin-2 cKO mice. To do this, lung tissues from the mice were collected and examined for the presence of lung metastases. Gross observation revealed that by 15 weeks of age, the PyMT; WT mice developed large numbers of metastatic lung nodules (Fig. 6A). In contrast, only a few metastatic surface nodules were detected in the PyMT; Kindlin-2 cKO mice (Fig. 6A). Lung histological staining further confirmed this observation, in which both the number and areas of metastatic nodules were remarkably reduced in the PyMT; Kindlin-2 cKO mice compared with those in the PyMT; WT mice (Fig. 6B–D). To test the role of Kindlin-2 in breast cancer metastasis directly, we employed an experimental metastasis mouse model in which Kindlin-2 was knocked down from BT549 cells by short hairpin RNA (shRNA)-based RNA interference (Fig. 6E) and then equal number of Kindlin-2 knockdown BT549 cells and the control BT549 cells, respectively, were injected into the mouse tail vein. Hematoxylin-eosin (H&E) analysis showed that depletion of Kindlin-2 from BT549 cells resulted in a significant reduction of the areas of metastatic nodules in the lung (Fig. 6F, G), suggesting an important role of Kindiln-2 in regulation of breast cancer metastasis.

## Discussion

**Fig. 7:**
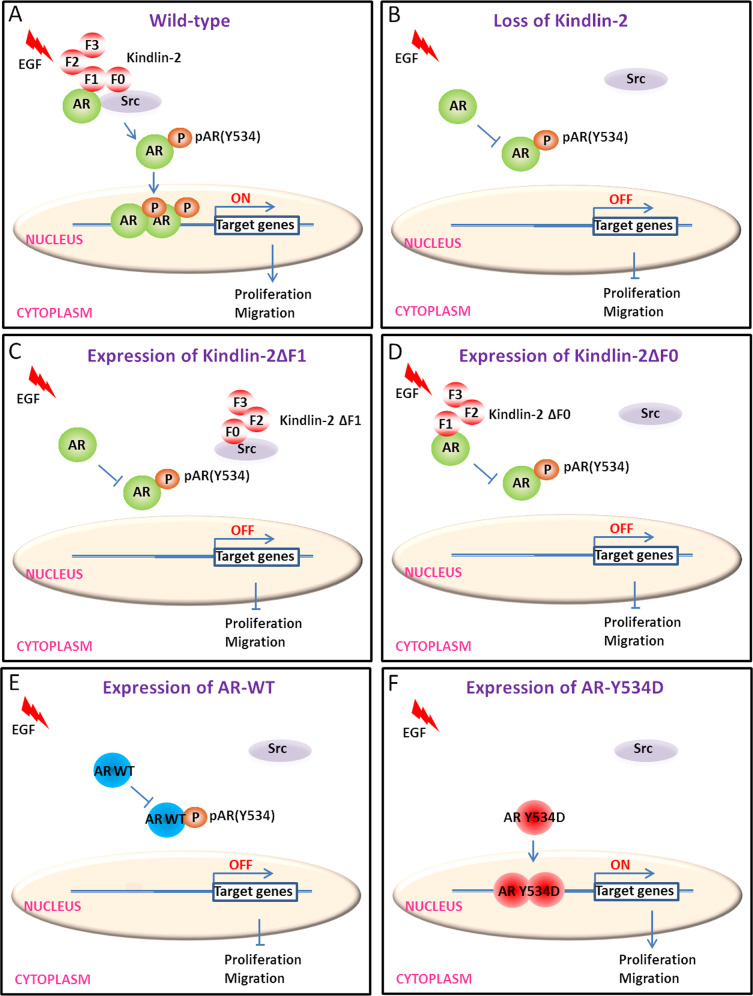
A working model of Kindlin-2-AR-Src signaling in breast cancer cells. The picture depicts a model in which the Kindlin-2-AR-Src complex delineated in the current study promotes AR Tyr-534 phosphorylation, downstream signaling, breast cancer cell proliferation and migration. **A** In wild-type breast cancer cells, Kindlin-2 acts as a scaffold to recruit Src and AR to form a supramolecular complex, and thereby facilitates Src-dependent AR Tyr-534 phosphorylation and promotes AR nuclear translocation in response to EGF stimulation, leading to increased expression of AR target genes, breast cancer cell proliferation and migration. **B** In Kindlin-2 deficient cells, loss of Kindlin-2 impairs the complex formation between Src and AR, resulting in diminished AR Tyr-534 phosphorylation, signaling, breast cancer cell proliferation and migration. In Kindlin-2 deficient cells expressing AR-binding-defective mutant (Kindlin-2-ΔF1) (**C**) or Src-binding-defective mutant (Kindlin-2-ΔF0) (**D**), the Kindlin-2 mutants are unable to promote the formation of a supramolecular complex containing both Src and AR, and therefore are unable to promote Src-dependent AR Tyr-534 phosphorylation, signaling, breast cancer cell proliferation and migration. **E**, **F** In Kindlin-2 deficient cells expressing phospho-mimic mutant of AR (AR Y534D) promotes AR nuclear translocation, and then increases AR target genes expression, breast cancer cell proliferation and migration (**E**). However, overexpression of wild-type AR (AR WT) in Kindlin-2 knockdown cells failed to do so (**F**).

AR is emerging as a promising prognostic biomarker and it may serve as a potential therapeutic target for treatment of breast cancer [11]. It has been shown that high level of AR expression are found in 70–90% breast cancer [11, 82]. In addition to elevated expression, increased AR tyrosine phosphorylation, activation and signaling are also critical for promotion of cancer progression [11, 13, 14, 82]. In the current study, we have demonstrated that Kindlin-2 is crucial for AR signaling and breast cancer progression. Furthermore, the findings obtained from this study shed important light on the molecular mechanism by which Kindlin-2 regulates this process. Using a photo-pTyr-scaffold screen, we have found that Kindlin-2 regulates AR Tyr-534 phosphorylation, an event that is critical for AR activation, downstream signaling, and cancer progression [13, 14, 72]. Furthermore, employing a combination of molecular and cellular approaches, we have shown that Kindlin-2 associates with AR through its F1 subdomain, which neighbors the Src-binding F0 subdomain, resulting in the formation of a supramolecular complex containing Kindlin-2, AR and Src. Finally, using a mutational strategy, we provide evidence showing that Kindlin-2-mediated associations with AR and Src are crucial for AR Tyr-534 phosphorylation, its downstream signaling, breast cancer cell proliferation and migration. Based on these findings, we propose a model (Fig. 7) in which Kindlin-2 acts as a key scaffolding protein to facilitate the association of Src with AR and consequently promote AR Tyr-534 phosphorylation and downstream signaling in response to extracellular stimuli such as EGF, resulting in increased cell proliferation, migration and breast cancer progression.

While our studies strongly suggest that Kindlin-2 regulates breast cancer progression through, at least in part, promotion of Src-mediated AR Tyr-534 phosphorylation, downstream signaling, our findings do not rule out the possibility that Kindlin-2 may participate in breast cancer progression through regulation of other pathways. For example, Kindlin-2 is known to play a critical role in regulation of cell-extracellular matrix adhesion, integrin signaling and metabolic reprogramming [36–47, 51, 55, 60–62, 67], which likely contribute to Kindlin-2 deficiency-induced inhibitory effects on breast cancer growth and metastasis described in the current study. The potent inhibitory effects of Kindlin-2 deficiency on breast cancer progression and its role in regulation of AR (the current study) as well as other signaling pathways [36–47, 51, 55, 60–62, 67], together with the fact that Kindlin-2 is dispensable for normal mammary gland development (Supplementary Fig. 3), suggest that targeting the Kindlin-2 signaling pathway may provide a useful approach for therapeutic control of breast cancer progression and thus may help to improve the clinical outcome of human patients with breast cancer.

Finally, although our current study focuses on breast cancer, abnormal AR signaling is known to be involved in malignancies in other organs (e.g., prostate) as well as several other pathological processes [83]. Thus, it will be interesting to investigate in future studies whether the Kindlin-2-Src-AR signaling axis delineated in the current study also plays a role in prostate cancer and other human diseases associated with aberrant AR signaling.

## Materials and methods

### Animal studies

Kindlin-2^fl/fl^ mice (C57BL/6 background) generated as described before [84] were backcrossed seven generations with FVB mice (from the Jackson Laboratory, Stock No. 001800) to produce Kindlin-2^fl/fl^ mice (FVB background). For selective deletion of Kindlin-2 in mammary gland epithelial cells, Kindlin-2^fl/fl^ mice (FVB background) were crossed with MMTV-Cre mice (FVB background, from the Jackson Laboratory, Stock No. 003553) to generate Kindlin-2^fl/fl^;MMTV-Cre (Kindlin-2 cKO) and Kindlin-2^+/+^;MMTV-Cre (Kindlin-2 WT). Kindlin-2 cKO and WT mice were further crossed with MMTV-PyMT mice (FVB background, from the Jackson Laboratory, Stock No.002374) to produce PyMT; Kindlin-2 cKO and PyMT;WT mice. Tail genotyping was performed by routine PCR protocol. PCR primers used for analyzing were listed: (1) Kindlin-2: 5′-GGCTCTTTCTACTTCTGTTCCT-3′, 5′-AACCAACCAACTAATCAGCCAG-3′; (2) Cre: 5′-ACGAGTGATGAGGTTCGCAAG-3′, 5′-CAATCCCCAGAAATG CCAGA-3′; (3) PyMT: 5′-AACGGCGGAGCGAGGAACTG-3′, 5′-ATCGGGCTCAGCAACACAAG-3′. Female PyMT; Kindlin-2 cKO and their wild-type littermates PyMT;WT mice were monitored for tumors by palpation every 2 days.

Pulmonary metastasis was induced by injecting 2 × 10^6^ BT549 cells into 5-week-old female BALB/c nude mice via tail vein. After 9 weeks, mice were euthanized and lungs were collected. Lung metastases were determined by H&E staining analysis.

All animal procedures were approved by the Institutional Animal Care and Use Committee at the Southern University of Science and Technology.

### Cell culture

Human BT549 cells and HEK 293T cells were obtained from American Type Culture Collection (ATCC). MDA-MB-453 cells were obtained from the National Collection of Authenticated Cell Cultures (Shanghai, China). BT549 cells and HEK239T were cultured in Dulbecco’s modified Eagle’s medium (DMEM) supplemented with 10% fetal bovine serum (FBS) (Gibco-Invitrogen), 50U/ml penicillin and streptomycin at 37 °C in 5% CO_2_. Prior to shipping each cell line, the ATCC performed cell line authentication and *mycoplasma* testing. MDA-MB-453 cells were cultured in Roswell Park Memorial Institute (RPMI) 1640 medium supplemented with 10% FBS (Gibco-Invitrogen), 50U/ml penicillin and streptomycin at 37 °C in 5% CO_2_. Cells were authenticated through the short tandem repeat analysis method and *mycoplasma* contamination was excluded.

### Reagents

Plasmid psPAX2, pMD2.G and pLVX-Flag-Hyg to produce lentivirus were obtained from Addgene. AR luciferase reporter AR-Luc (HZBIO, Cat# 11636ES70) was obtained from HZBIO, China. Full-length AR cDNA was obtained from Vigene Biosciences, Cat# NM-000044.

Antibodies against Kindlin-2 (ProteinTech, Cat#11453-1-AP), Src (Cell Signaling Technology, Cat#2109S), AR (Cell Signaling Technology, Cat#5153S), phospho-Y534 AR (Invitrogen, Cat#PA5-64643), phospho-Y267 AR (Rockland, Cat#600-401-j95), α-tubulin (Sigma-Aldrich, Cat# T6074), Flag (Sigma-Aldrich, Cat# F1804), cyclin D1 (Abcam, Cat# ab16663) and GST (Transgen Biotech, Cat# HT601-02) were purchased. Antibody against Kindlin-2 for IP was from Millipore (clone 3A3, Millipore, Cat# MAB2617). Mouse control IgG (Santa Cruz biotechnology, Cat#sc-2025) and rabbit control IgG (Invitrogen, Cat#31235) was purchased.

Hematoxylin (Baso, Cat# BA4097) and eosin (Baso, Cat# BA4098) for H&E staining were purchased. Carmine Alum (Sigma, Cat#C1022) for whole mount staining and propidium iodide (Sigma, Cat# P4170) for flow cytometry was obtained from Sigma. Tripure isolation reagent (Roche, Cat#11667165001), ReverTra Ace® qPCR RT Master Mix (TOYOBO, Cat# FSQ-201) and LightCycler® 480 SYBR Green I Master (Roche, Cat# 04887352001) for qPCR were purchased. Cell lysis buffer for IP (Beyotime, Cat# P0013) was obtained from Beyotime, China. Protease inhibitor cocktail (Med Chem Express, Cat# HY-K0010) and phosphatase inhibitor cocktail (Bimake, Cat# B15001) were purchased. RNase (Thermo Fisher Scientific, Cat# EN0531) was obtained from Thermo Fisher Scientific. EGF (R&D System, Cat# PRD236-50) was purchased. Dual-Luciferase® Reporter Assay System (Promega, Cat# E1960) was obtained from Promega.

### RNAi

Kindlin-2 siRNA target sequences: sense, 5′-CAGCGAGAAUCUUGGAGGCTT-3′; antisense, 5′-GCCUCCAAGAUUCUCGCUGUU-3′. Negative control siRNA: sense, 5′-UUCUCCGAACGUGUCACGUTT-3′; antisense, 5′-ACGUGACACGUUCGGAGAATT-3′. All siRNAs were purchased from Gene Pharma.

### Whole mount staining

Mouse mammary fat pads were surgically collected, spread onto a glass slide, and fixed with Carnoy’s fixative (1:3 mixture of glacial acetic acid and 100% ethanol) overnight. After hydration, mammary fat pads were stained with Carmine Alum overnight, then dehydrated and cleared in xylenes and finally mounted with Permount (Thermo Scientific, Cat# SP15-100). Stained slides were scanned with a digital camera (DS-Fi1c; Nikon) and NIS-Elements F Ver4.30.01 image analysis software (Nikon). Hyperplasia lesions in mammary glands were analyzed using Image J (NIH, USA).

### Immunohistochemical (IHC) staining of paraffin-embedded tissue slides

Four µm-thick tissue sections were deparaffinized. Antigen retrieval was done using pH 8.0 10 nM sodium citrate solution at 100 °C for 15 min. Immunohistochemical staining was performed using primary antibodies as specified in each experiment at 4 °C overnight. All stained slides were scanned using Leica SCN400 slide scanner and the images were analyzed using Image-Pro Plus software version 6 (Media Cybernetics, Silver Spring, MD).

### Total protein extraction from mammary gland

To extract total proteins from mammary tumor tissues, mouse mammary glands were collected from 8-week-age mice. After removal of lymph nodes, the mammary tissues were cut into 0.1-mm^3^ pieces on ice and digested in 1 mg/ml collagenase I (Worthington Biochemical, Cat# LS004196) for 2 h at 37 °C with gentle shaking to remove the adipose tissue. After digestion, the mammary tissues were collected by 5 min of centrifugation at 1000 × *g* and washed three times with 1 ml of 1 × PBS. Cell lysis buffer (Beyotime, Cat# P0013) containing protease inhibitor and phosphatase inhibitor was then added to lyse the tissues. Subsequently, samples were processed for immunoblotting.

### Lentiviral infection

To generate various Kindlin-2 or AR overexpression cell lines, pLVX-Kindlin-2-WT-Hyg, pLVX-Kindlin-2-ΔF0-Hyg, pLVX-Kindlin-2-ΔF1-Hyg, pLVX-AR-WT-Hyg, pLVX-AR-Y534D-Hyg or pLVX-Flag-Hyg were co-transfected with psPAX2 and pMD2.G into HEK 293T cells. The culture media containing the lentivirus were collected on the 3rd day after transfection, filtered (pore size 45 µm) and concentrated by ultracentrifugation (50,000 × *g*). The concentrated virus soup was immediately used or stored at −80 °C. For lentiviral infection, BT549 cells were cultured in growth media until 50% confluence and then replaced with fresh media containing lentivirus at a multiplicity of infection (MOI) of 100 mixed with 8 μg/ml polybrene for 16 h. The viral infection efficiency was confirmed by immunoblotting

### Immunofluorescence staining

To monitor AR nuclear translocation, cells were treated using serum-free media for 12 h (BT549 cells) or for 24 h (MDA-MB-453 cells) and then were added 100 ng/ml EGF (R&DSystems, Cat#PRD236) or vehicle for another 12 h. After that, cells were fixed with 4% paraformaldehyde, permeabilized by 0.1% Triton X-100 in 1×PBS, blocked by 5% BSA in room temperature and incubated with anti-AR antibody at 4 °C overnight. Cells were then stained by Alexa Fluor 488-conjugated IgG and mounted by mounting media with 4,6-diamidino-2-phenylindole. Fluorescence microscope (Nikon Confocal A1R with FLIM) was used to visualize stained cells.

### Immunoblotting analysis

The cells were lysed in 1 × sodium dodycyl sulfate (SDS) buffer containing protease inhibitor. Proteins were analyzed by immunoblotting as described before [85].

For AR phosphorylation analysis, cells were cultured in serum-free DMEM or PRIM 1640 media for 24 h. In total, 100 ng/ml EGF was added into media and cells were incubated at 37 °C for 5 min. In total, 1 × SDS buffer containing protease inhibitor and phosphatase inhibitor was used to lyse the cells.

### Co-immunoprecipitation

The cells were lysed with IP lysis buffer (Beyotime, Cat# P0013) containing 1 mM phenylmethylsulfonyl fluoride (PMSF). After incubating on ice for 30 min and centrifuging for 15 min at 4 °C, the supernatants were collected. Equal amounts of total lysates (3 mg) were incubated overnight at 4 °C with 30 µl of protein A/G-Sepharose beads. In parallel, 50 µl of protein A/G-Sepharose beads were incubated for 2 h at 4 °C with antibodies of interest (2 µg) to generate the immunobeads, which were subsequently mixed with lysates and incubated overnight at 4 °C. The next day, beads were rinsed three times in 1 × PBS with 1 mM PMSF. Proteins were eluted from Sepharose beads by mixing with 60 µl of 1 × SDS polyacrylamide gel electrophoresis loading buffer (containing 10% β-mercaptoethanol). Subsequently, samples were processed for immunoblotting.

### GST pull-down assay

For generation of GST-fusion proteins containing full-length or various mutants of Kindlin-2, cDNAs encoding Kindlin-2 or its fragments were cloned into pGEX-4T-1 vector. *Escherichia coli* strain BL21 were then transformed with the expression vectors. GST and GST-fusion proteins were purified from *E.coli* BL21 using Glutathione-Sepharose 4B matrix (GE Healthcare) according to the manufacturer’s instructions. Purified proteins were resolved by SDS-PAGE to verify their size and purity. In pull-down assays, GST or GST-fusion proteins were bound to Glutathione-Sepharose, mixed with BT549 cell lysates and incubated overnight at 4 °C. Subsequently, the beads were washed three times with 1 ml of 1 × PBS containing 0.2% Triton X-100. GST and GST-fusion proteins bound to the beads were eluted and analyzed by immunoblotting.

### Cell cycle assay

To monitor cell cycle, cells were treated by serum-free media with 100 ng/ml EGF or vehicle reagent. After 72 h, cells were fixed using 75% ethanol and stained with propidium iodide. Cells were detected by using a FACS cytometer (BD FACSCanto™) and analyzed with FlowJo software.

### Cell migration assay

Transwell cell migration were performed using cell culture inserts with 8.0-µm-pore size membranes (Corning, Cat#3422) following the manufacturer’s protocol. Cells (2 × 10^4^) were seeded onto the top chamber with 2% charcoal-stripped FBS containing DMEM or PRIM 1640 media. In the bottom chamber, either 100 ng/ml EGF or vehicle in 2% charcoal-stripped FBS containing DMEM media was added as a chemoattractant. After 12 h incubation, cells migrating to undersurface of the chamber were fixed by 4% paraformaldehyde for 30 min and were stained by Hochest 33342 for 30 min. Migrated cells were counted under inverted microscope (Nikon T1-SAM).

### Cell proliferation assay

BT549 cells (6 × 10^4^) were cultured in serum-free DMEM media containing 100 ng/ml EGF or vehicle. MDA-MB-453 cells (2 × 10^5^) were cultured in serum-free RPMI 1640 media containing 100 ng/ml EGF or vehicle. After 72 h, the cells were collected and counted using CountStar.

### RT-qPCR

Human BT549 or MDA-MB-453 cells were cultured in serum-free DMEM or RPMI 1640 media containing 100 ng/ml EGF or vehicle for 24 h. Total RNA was extracted using Tripure isolation reagent (Roche, Cat#11667165001) following the manufacturer’s instructions. Reverse transcription reactions were carried out with ReverTra Ace® qPCR RT Master Mix (TOYOBO, Cat# FSQ-201). Real-time PCR reactions used LightCycler® 480 SYBR Green I (Roche, Cat# 04887352001). As a control, the level of GAPDH mRNA was quantified in parallel with mRNAs of the target genes. Primers used for RT-qPCR were:

1. GAPDH: 5′-GTGAAGGTCGGAGTCAACGG-3′ and 5′-TCCTGGAAGATGG TGATGGG-3′.
2. Cyclin D1: 5′-ATCAAGTGTGACCCGGACTG-3′ and 5′-CTTGGGGTCCATG TTCTGCT-3′.

### Luciferase assay

In brief, AR luciferase reporter vector (HZBIO, Cat# 11636ES70) was co-transfected with psPAX2 and pMD2.G into HEK 293T cells. The culture media containing the lentivirus was harvested on the 3rd day after transfection. For lentiviral infection, BT549 and MDA-MB-453 cells were cultured in growth media until 50% confluence and then replaced with fresh media containing lentivirus at a MOI of 100 mixed with 8 μg/ml polybrene for 16 h. AR-luc expressing cells were then treated using serum-free media with 100 ng/ml EGF or vehicle for 24 h. Cells were processed using Dual-Luciferase® Reporter Assay System. Luciferase activities were quantified by microplate reader (EnSpire).

### Photo-pTyr-scaffold pull-down and MS analysis

Control or Kindlin-2 knockdown BT549 cells were cultured in serum-free DMEM media containing 100 ng/ml EGF or vehicle for 24 h. Cells were lysed by ice-cold NP-40 buffer (50 mM Tris-HCl, 150 mMNaCl, and 1% (v/v) NP-40, pH 7.4). The Photo-pTyr-scaffold operation was performed as described before [86]. In brief, the obtained cell lysates were sonicated for 5 s at 200 W 4 times with 10 s rest for each time. The lysate was cleaned up by centrifugation at 14,000 × *g* for 10 min at 4 °C. The protein concentration was measured by BCA assay (Thermo Fisher Scientific). Fifty micrograms of Photo-pTyr-scaffold was incubated with the freshly isolated total lysate in NP-40 buffer. Samples were incubated for 2 h at 4 °C with moderate shaking followed by 30 min UV irradiation (UVP CL-1000L UV Crosslinker, 365 nm). The samples were then incubated with 30 μl of streptavidin beads (GE Healthcare) for 2 h at 4 °C. After incubation, samples were washed three times with harsh modified RIPA buffer [50 mM Tris-HCl, 1 M NaCl, 1% (v/v) Triton X-100, 1% (w/v) sodium deoxycholate, and 1% (w/v) SDS, pH 7.4] and one time with 50 mM ammonium bicarbonate (ABC). After being reduced by 10 mM Tris (2-carboxyethyl) phosphine hydrochloride (TCEP) and alkylated by 30 mM iodoacetamide (IAA), the beads were washed with 50 mM ABC and incubated with trypsin (Promega) overnight at 37 °C. The digested peptides were washed with 1% (v/v) formic acid, subjected to StageTip C18 desalting and MS analysis.

The raw data were searched with MaxQuant software (version 1.5.5.1) against the human UniProt FASTA database (70,956 entries, downloaded on Dec 9, 2016). The false discovery rate evaluation was done by searching a reverse database and was set to 0.05 for proteins and peptides. All statistical and bioinformatics analyses were performed with the Perseus software (version 1.5.5.3) and Microsoft Excel.

### Statistical analyses

All data represent as mean ± SEM. Two-tailed Student’s *t* test was used to compare two groups of samples. One-way ANOVA was used for multiple comparisons. Survival analysis was carried out using the log-rank test. *p* values <0.05 were considered significant. Prism 7 (GraphPad) was used for statistical analysis.

## Supplementary information

Supplementary Figures-clean version

Supplementary Table S1

Supplementary Original Western Blots

Reproducibility checklist

## Data Availability

All data generated or analyzed during this study are included in this published article and its Supplementary files. Requests for materials should be addressed to YS.
