# Supplementary material for: Kindlin-2 promotes Src-mediated tyrosine phosphorylation of androgen receptor and contributes to breast cancer progression
Source: Cell Death Dis. 2022 May 20;13(5):482. doi: 10.1038/s41419-022-04945-z (PMC9122951; doi:10.1038/s41419-022-04945-z)
Supplement: Supplementary file 1 — Supplementary Figures-clean version [file 41419_2022_4945_MOESM1_ESM.docx]

**
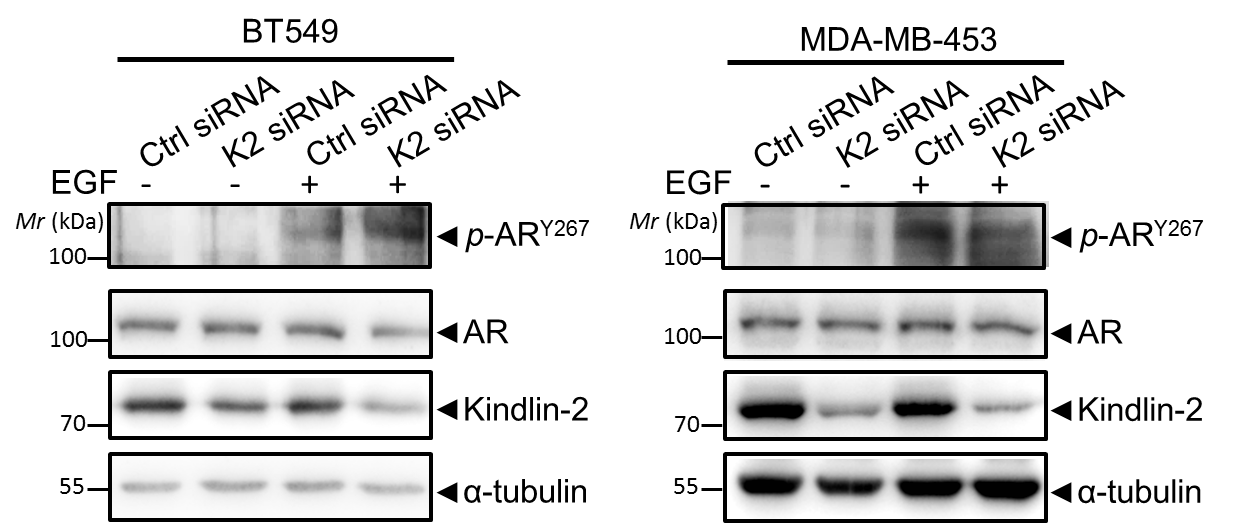
**

**Supplementary Figure 1. Depletion of Kindlin-2 did not affect AR Tyr-267 phosphorylation.** Immunoblotting analysis of total AR and AR Tyr-267 phosphorylation level in control (Ctrl siRNA) and Kindlin-2 knockdown (K2 siRNA) BT549 cells or MDA-MB-453 cells with or without EGF stimulation.


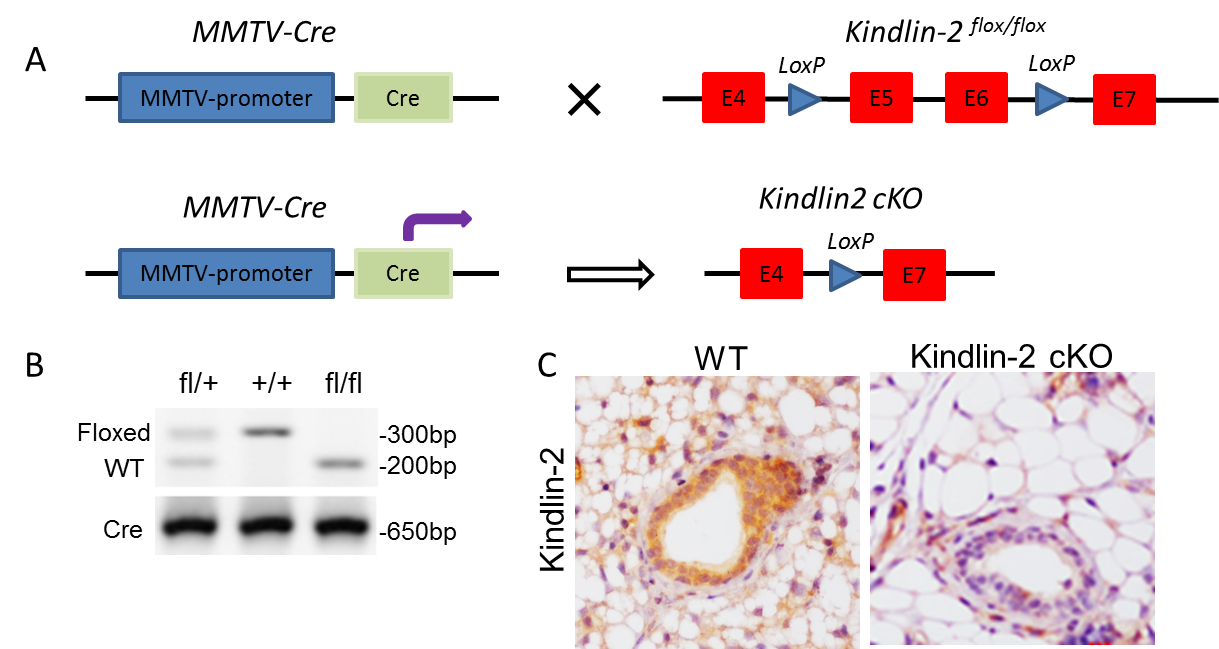


**Supplementary Figure 2. Generation of mammary epithelium-specific Kindlin-2 knockout mice (Kindlin-2 cKO mice). (A)** The diagram depicts the strategy for generation of Kindlin-2 cKO mice. Mice expressing MMTV-Cre were bred with mice carrying floxed Kindlin-2 locus (exons 5 and 6). **(B)** Representative PCR analysis of extracted genomic DNA from tail clippings. PCR product bands of floxed (300 bp) and wild-type (200 bp) were shown. Cre PCR product (650 bp) was also indicated. **(C)** Immunohistochemistry (IHC) analysis of Kindlin-2 expression in mammary glands from wild-type (WT) and Kindlin-2 cKO littermates. Original magnification, ×400.


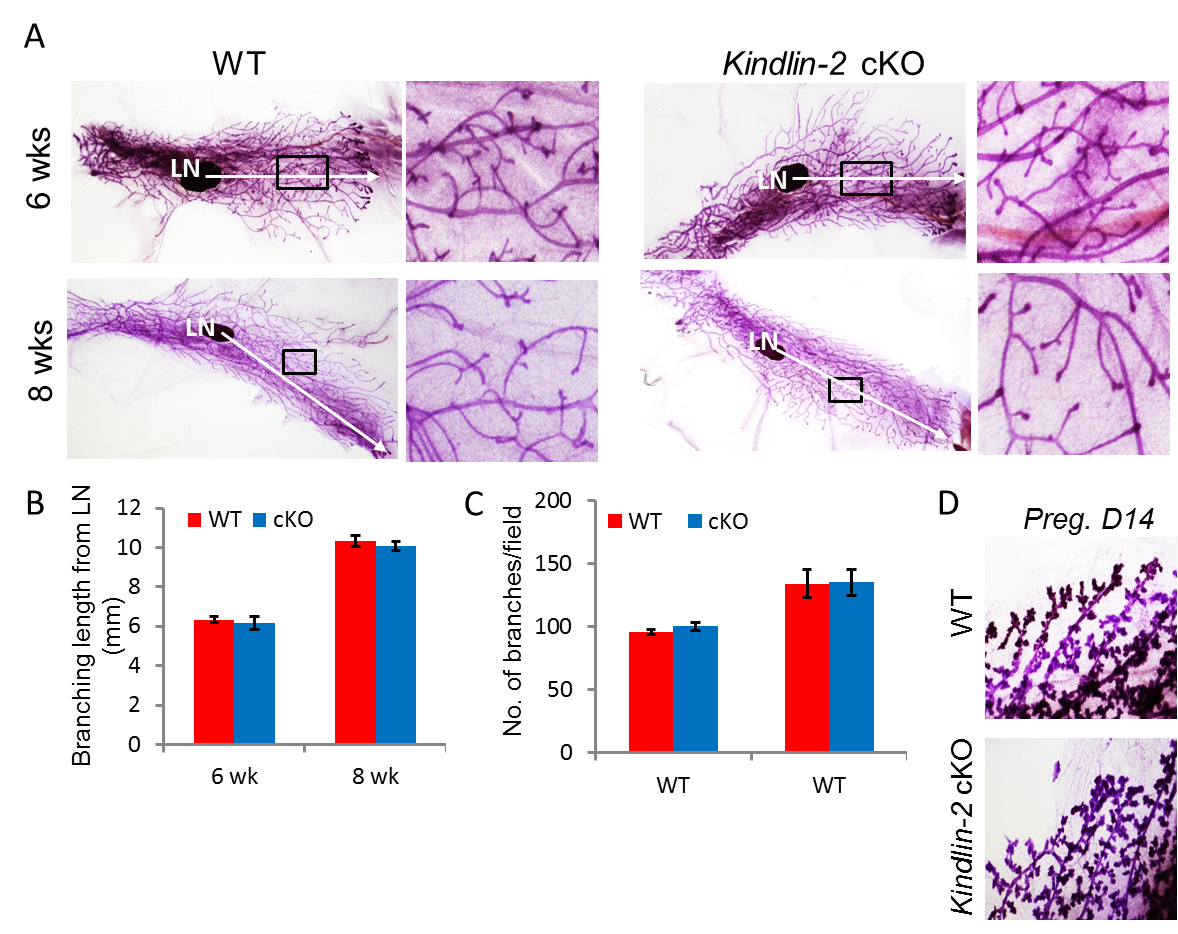


**Supplementary Figure 3. Loss of Kindlin-2 did not affect normal mammary gland development. (A)** Representative images of mammary gland whole-mounts from wild-type (WT) and Kindlin-2 cKO littermates with indicated ages. LN: lymph node. Original magnification, ×6.3. Higher magnification images of representative areas of whole-mount staining (indicated by squares) were shown on the right panels. **(B)** Quantification of the length of epithelial tree from lymph node in the mammary glands of WT and Kindlin-2 cKO littermates with indicated ages. *n* = 3 mice at each time point. **(C)** Quantification of the mean number of branches in the mammary glands of WT and Kindlin-2 cKO littermates with indicated ages. *n* = 3 mice at each time point. **(D)** Representative images of mammary gland whole-mounts from WT and Kindlin-2 cKO littermates at pregnant day 14. Original magnification, ×60.
