# Supplementary material for: Kindlin-2 promotes Src-mediated tyrosine phosphorylation of androgen receptor and contributes to breast cancer progression
Source: Cell Death Dis. 2022 May 20;13(5):482. doi: 10.1038/s41419-022-04945-z (PMC9122951; doi:10.1038/s41419-022-04945-z)
Supplement: Supplementary file 2 — Supplementary Table S1 [file 41419_2022_4945_MOESM2_ESM.docx]

**Supplementary Table S1.** Photo-pTyr-scaffold approach enriched proteins

[List in descending order of enrichment difference (Ctrl siRNA+EGF/K2 siRNA+EGF)]

| Filamin-C |
| --- |
| S-formylglutathione hydrolase |
| Solute carrier family 35 member F6 |
| Elongation factor 1-alpha 2 |
| Lipoma HMGIC fusion partner-like 2 protein |
| Histone-lysine N-methyltransferase SETDB1 |
| Fermitin family homolog 2 |
| Transmembrane protein 41B |
| Nidogen-2 |
| Thrombospondin-3 |
| Eukaryotic translation initiation factor 4B |
| NADH-ubiquinone oxidoreductase chain 2 |
| Microtubule-associated protein 2 |
| Synaptogyrin-3 |
| Vesicle-associated membrane protein 7 |
| Monocarboxylate transporter 7 |
| Vacuolar protein sorting-associated protein 13B |
| Transmembrane protein 126A |
| Collagen alpha-1(XIX) chain |
| DNA replication licensing factor MCM6 |
| Integrin beta-4 |
| Carnitine O-palmitoyltransferase 1, brain isoform |
| Rho GTPase-activating protein 24 |
| Angiomotin-like protein 1 |
| 40S ribosomal protein S26;Putative 40S ribosomal protein S26-like 1 |
| Anion exchange protein |
| Synaptopodin |
| Glutaredoxin-1 |
| Ceroid-lipofuscinosis neuronal protein 6 |
| NADH dehydrogenase [ubiquinone] 1 subunit C2;NADH dehydrogenase [ubiquinone] 1 subunit C2, isoform 2 |
| Rho GTPase-activating protein 42 |
| Adenylate kinase isoenzyme 6 |
| Lipid phosphate phosphohydrolase 3 |
| F-box only protein 22 |
| Protein YIPF5;Protein YIPF |
| Serine/threonine-protein kinase WNK1 |
| Fibrosin-1-like protein |
| Dedicator of cytokinesis protein 9 |
| Putative Dol-P-Glc:Glc(2)Man(9)GlcNAc(2)-PP-Dol alpha-1,2-glucosyltransferase;Dol-P-Glc:Glc(2)Man(9)GlcNAc(2)-PP-Dol alpha-1,2-glucosyltransferase |
| Snurportin-1 |
| Integrator complex subunit 8 |
| YTH domain-containing protein 1 |
| Opioid growth factor receptor |
| Folate transporter 1 |
| Pyridoxal-dependent decarboxylase domain-containing protein 1 |
| RNA polymerase-associated protein CTR9 homolog |
| Protein FAM162A |
| Ras-related GTP-binding protein A;Ras-related GTP-binding protein B |
| Urea transporter 1 |
| Delta(14)-sterol reductase |
| DNA repair protein RAD51 homolog 1 |
| Multiple myeloma tumor-associated protein 2 |
| Vacuolar protein sorting-associated protein 41 homolog |
| ATP synthase subunit e, mitochondrial |
| Docking protein 1 |
| Peroxisomal membrane protein 11B |
| Target of Myb protein 1 |
| Enhancer of mRNA-decapping protein 3 |
| Nitric oxide synthase-interacting protein |
| CD320 antigen |
| Transcriptional repressor p66-beta |
| GRAM domain-containing protein 1B |
| Solute carrier organic anion transporter family member 4A1 |
| Protein-glutamine gamma-glutamyltransferase 2 |
| Phosphorylated adapter RNA export protein |
| Single-stranded DNA-binding protein 4 |
| Proline-rich basic protein 1 |
| Testis-expressed sequence 10 protein |
| Golgi phosphoprotein 3 |
| 60S ribosomal protein L7a |
| Ribonucleases P/MRP protein subunit POP1 |
| Conserved oligomeric Golgi complex subunit 4 |
| Complement component 1 Q subcomponent-binding protein, mitochondrial |
| Frizzled-6 |
| Serine/threonine-protein phosphatase 6 regulatory subunit 2 |
| Matrix-remodeling-associated protein 8 |
| Fucose-1-phosphate guanylyltransferase |
| Ribonucleoside-diphosphate reductase large subunit |
| Androgen receptor |
| DBIRD complex subunit ZNF326 |
| Liprin-alpha-1;Liprin-alpha-2 |
| Smoothelin |
| Ras GTPase-activating-like protein IQGAP2 |
| AarF domain-containing protein kinase 4 |
| Protein shisa-2 homolog |
| Protein BANP |
| Histidine--tRNA ligase, cytoplasmic |
| tRNA (guanine(10)-N2)-methyltransferase homolog |
| Oxysterol-binding protein-related protein 9 |
| DNA polymerase;DNA polymerase delta catalytic subunit |
| Ceramide synthase 6 |
| Leucine-rich repeat-containing protein 57 |
| Protein FAM65A |
| ESF1 homolog |
| Transcription cofactor vestigial-like protein 3 |
| Frizzled-2 |
| Ubiquitin-fold modifier 1 |
| MAP7 domain-containing protein 1 |
| SLIT-ROBO Rho GTPase-activating protein 1 |
| Integrin-linked kinase-associated serine/threonine phosphatase 2C |
| Serine/threonine-protein phosphatase;Serine/threonine-protein phosphatase 5 |
| Ankyrin repeat domain-containing protein 50 |
| Rap guanine nucleotide exchange factor 1 |
| Pleckstrin homology-like domain family A member 2 |
| Calcium/calmodulin-dependent protein kinase type IV |
| Dual specificity protein phosphatase 3 |
| Exocyst complex component 7 |
| Tetratricopeptide repeat protein 13 |
| SCL-interrupting locus protein |
| DENN domain-containing protein 2A |
| Rab GDP dissociation inhibitor alpha |
| DNA damage-binding protein 1 |
| Chromatin complexes subunit BAP18 |
| CCR4-NOT transcription complex subunit 10 |
| Transcription initiation factor IIB |
| Transmembrane protein 192 |
| Glycogen synthase kinase-3 alpha |
| Spermatogenesis-associated protein 5-like protein 1 |
| Transcription initiation factor TFIID subunit 6 |
| Myotubularin-related protein 1 |
| Exostosin-like 3 |
| Serine/threonine-protein kinase DCLK1 |
| Nesprin-3 |
| Melanophilin |
| Cysteine and glycine-rich protein 1 |
| von Willebrand factor A domain-containing protein 8 |
| Transmembrane protein 97 |
| Calcium-activated potassium channel subunit alpha-1 |
| Dynein heavy chain 17, axonemal |
| Receptor-type tyrosine-protein phosphatase gamma |
| Nuclear receptor subfamily 0 group B member 1 |
| Palmdelphin |
| 7SK snRNA methylphosphate capping enzyme |
| Mitochondrial 10-formyltetrahydrofolate dehydrogenase |
| Protein THEMIS2 |
| Volume-regulated anion channel subunit LRRC8D |
| Nicotinamide N-methyltransferase |
| Serine/threonine-protein kinase SIK3 |
| Paraplegin |
| Uncharacterized protein KIAA1671 |
| NEDD4-binding protein 2-like 2 |
| Palladin |
| Cytospin-A |
| Cytochrome P450 1B1 |
| Copine-7 |
| DNA polymerase delta subunit 3 |
| Mannose-P-dolichol utilization defect 1 protein |
| Discoidin domain-containing receptor 2 |
| Ankyrin repeat domain-containing protein 10 |
| Focadhesin |
| Lymphokine-activated killer T-cell-originated protein kinase |
| Integrator complex subunit 5 |
| Nuclear pore membrane glycoprotein 210 |
| Ski oncogene |
| Serine/threonine-protein phosphatase 2A 65 kDa regulatory subunit A beta isoform |
| Kinesin-like protein KIF2A |
| Nuclear factor 1;Nuclear factor 1 B-type |
| T-box transcription factor TBX3 |
| Zinc finger CCCH-type antiviral protein 1-like |
| Regulation of nuclear pre-mRNA domain-containing protein 1A |
| Nucleolar protein 3 |
| Synaptotagmin-11 |
| Protein FAM83G |
| Eukaryotic translation initiation factor 1;Eukaryotic translation initiation factor 1b |
| Zinc finger CCCH domain-containing protein 14 |
| Nuclear autoantigenic sperm protein |
| Absent in melanoma 1 protein |
| Amyloid-like protein 1;C30 |
| Ras GTPase-activating protein nGAP |
| Asparagine--tRNA ligase, cytoplasmic |
| G patch domain and KOW motifs-containing protein |
| Echinoderm microtubule-associated protein-like 1 |
| Transmembrane protein 161B |
| Histone H4 |
| Protein SOGA1;N-terminal form;C-terminal 80 kDa form |
| L-aminoadipate-semialdehyde dehydrogenase-phosphopantetheinyl transferase |
| Mitochondrial ornithine transporter 1 |
| HCLS1-associated protein X-1 |
| Probable tRNA N6-adenosine threonylcarbamoyltransferase |
| Helicase POLQ-like |
| Hermansky-Pudlak syndrome 3 protein |
| Adenylosuccinate lyase |
| Multiple PDZ domain protein |
| Putative RNA polymerase II subunit B1 CTD phosphatase RPAP2 |
| Centlein |
| Endothelin-converting enzyme 1 |
| DNA mismatch repair protein Msh3 |
| Peroxisomal 2,4-dienoyl-CoA reductase |
| Interferon gamma receptor 1 |
| WD repeat-containing protein 18 |
| Forkhead box protein C1 |
| Protein SCO2 homolog, mitochondrial |
| Lysophospholipid acyltransferase 5 |
| 3-ketoacyl-CoA thiolase, peroxisomal |
| DmX-like protein 2 |
| Protein FAM168A |
| Cytochrome c oxidase protein 20 homolog |
| Vesicular integral-membrane protein VIP36 |
| 40S ribosomal protein S7 |
| cTAGE family member 5;Melanoma inhibitory activity protein 2;cTAGE family member 2 |
| Saccharopine dehydrogenase-like oxidoreductase |
| Transmembrane emp24 domain-containing protein 2 |
| E3 ubiquitin-protein ligase Midline-1 |
| Nucleolar protein 9 |
| Rho guanine nucleotide exchange factor 12 |
| Arf-GAP with Rho-GAP domain, ANK repeat and PH domain-containing protein 1 |
| Probable glutamate--tRNA ligase, mitochondrial |
| Keratinocyte-associated transmembrane protein 2 |
| Metastasis-associated protein MTA2 |
| Transducin-like enhancer protein 1;Transducin-like enhancer protein 4;Transducin-like enhancer protein 2;Transducin-like enhancer protein 3 |
| Protein prune homolog 2 |
| UPF0687 protein C20orf27 |
| Lariat debranching enzyme |
| ZZ-type zinc finger-containing protein 3 |
| Kinesin-like protein KIF11 |
| Cytoplasmic polyadenylation element-binding protein 4 |
| Palmitoyltransferase ZDHHC17 |
| AH receptor-interacting protein |
| Replication protein A 70 kDa DNA-binding subunit;Replication protein A 70 kDa DNA-binding subunit, N-terminally processed |
| Opioid growth factor receptor-like protein 1 |
| F-box only protein 44 |
| Nuclear factor NF-kappa-B p105 subunit;Nuclear factor NF-kappa-B p50 subunit |
| WD repeat domain phosphoinositide-interacting protein 4 |
| Holliday junction recognition protein |
| Serine-protein kinase ATM |
| WD repeat-containing protein WRAP73 |
| RNA polymerase II-associated factor 1 homolog |
| Transcription factor RelB |
| Mitochondrial chaperone BCS1 |
| Tumor necrosis factor receptor superfamily member 1A;Tumor necrosis factor receptor superfamily member 1A, membrane form;Tumor necrosis factor-binding protein 1 |
| Protein jagunal homolog 1 |
| Atrophin-1 |
| Protein VAC14 homolog |
| E3 ubiquitin-protein ligase TRIM56 |
| KN motif and ankyrin repeat domain-containing protein 1 |
| STE20-like serine/threonine-protein kinase |
| Cell division cycle protein 23 homolog |
| Integrator complex subunit 4 |
| Zinc finger protein 318 |
| Fibronectin;Anastellin;Ugl-Y1;Ugl-Y2;Ugl-Y3 |
| Tubulin beta-4A chain |
| Wings apart-like protein homolog |
| Testis-specific Y-encoded-like protein 2 |
| Integrator complex subunit 10 |
| Centrosomal protein of 170 kDa |
| Lysosomal-associated transmembrane protein 4A |
| Keratinocyte proline-rich protein |
| Neuron navigator 1 |
| Fibrillin-1 |
| Myotubularin-related protein 6 |
