# Supplementary material for: Kindlin-2 promotes Src-mediated tyrosine phosphorylation of androgen receptor and contributes to breast cancer progression
Source: Cell Death Dis. 2022 May 20;13(5):482. doi: 10.1038/s41419-022-04945-z (PMC9122951; doi:10.1038/s41419-022-04945-z)
Supplement: Supplementary file 3 — Supplementary Original Western Blots [file 41419_2022_4945_MOESM3_ESM.pdf]

Kindlin-2 promotes Src-mediated tyrosine phosphorylation of androgen receptor and contributes to breast cancer progression

Luyao Ma[1#], Yeteng Tian[1#], Tao Qian[1], Wenjun Li[1], Chengmin Liu[1], Bizhu Chu[2], Qian Kong[2], Renwei Cai[1], Panzhu Bai[1], Lisha Ma[1], Yi Deng[1], Ruijun Tian[2], Chuanyue Wu[3*], Ying Sun[1*]

Supplementary Original Western Blots

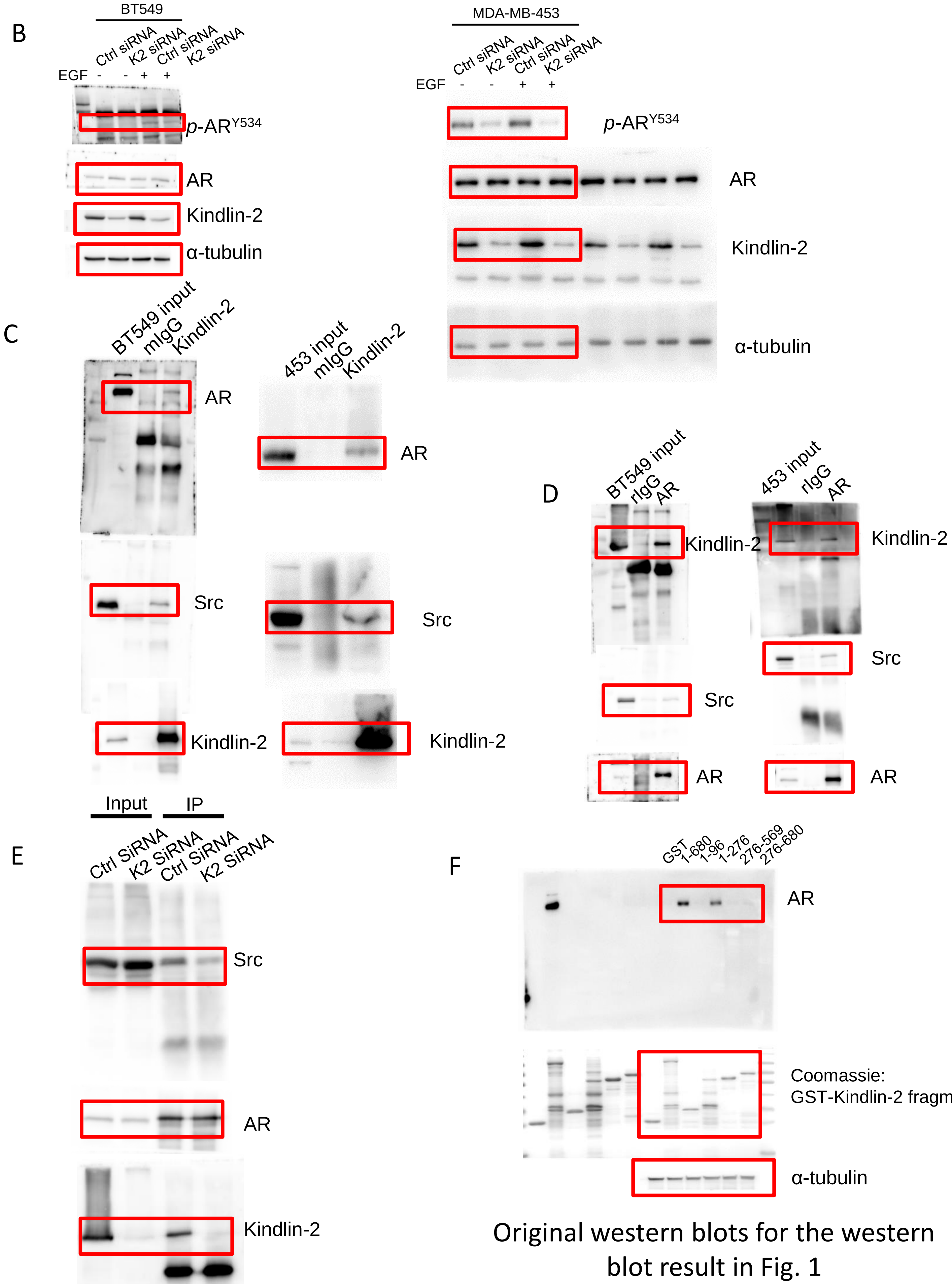

Original western blots for the western blot result in Fig. 1

D

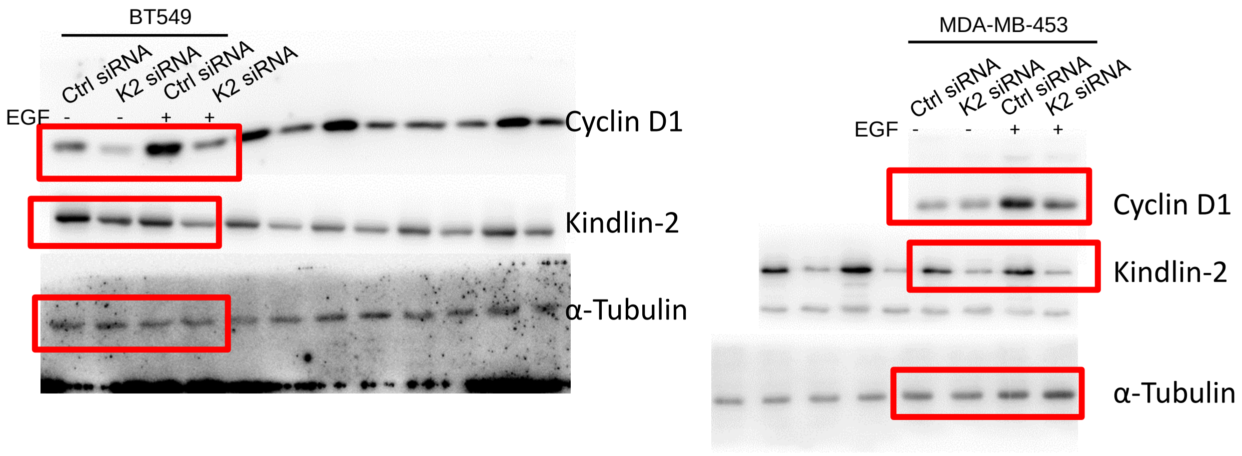

Original western blots for the western blot result in Fig. 2

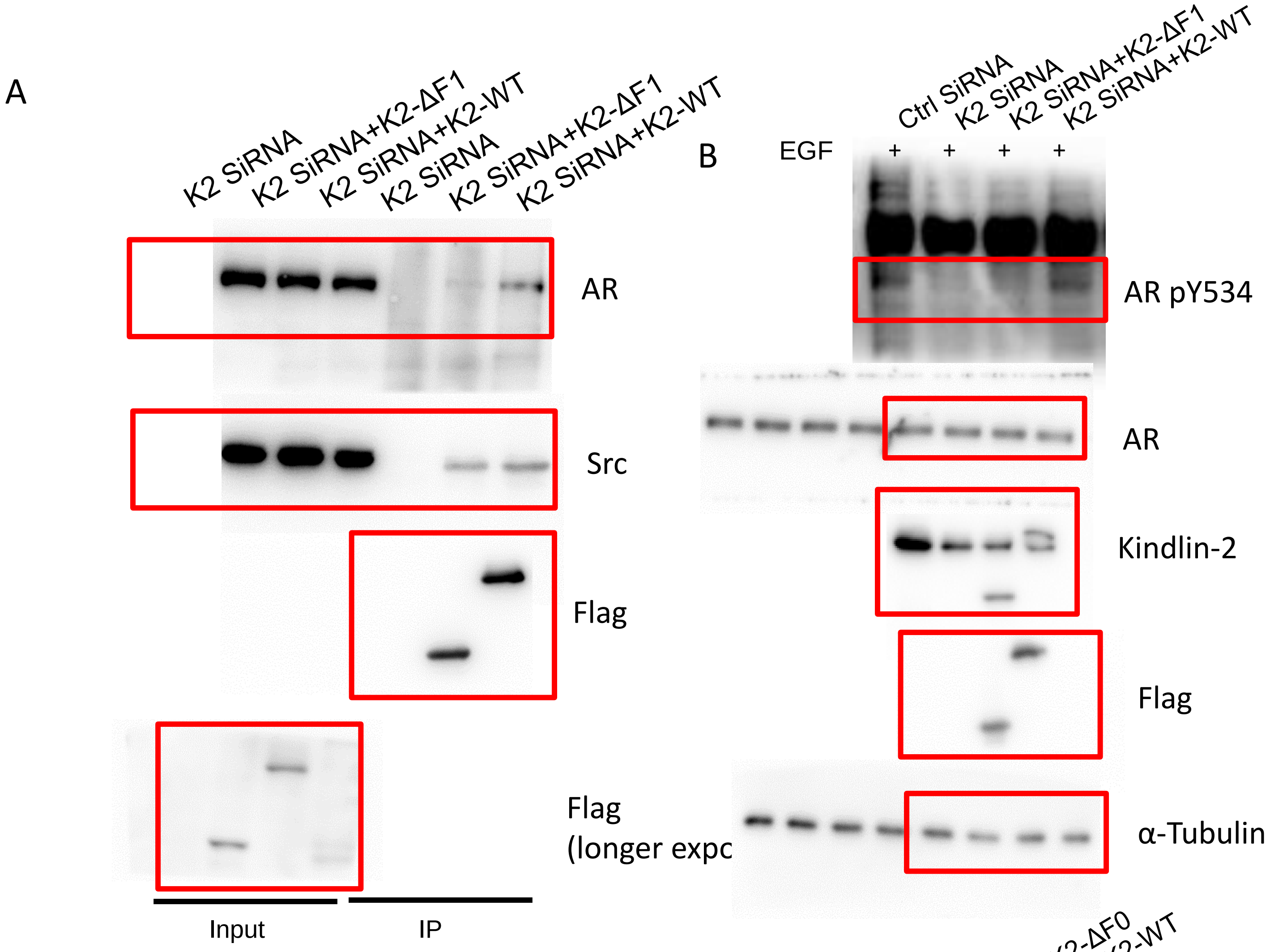

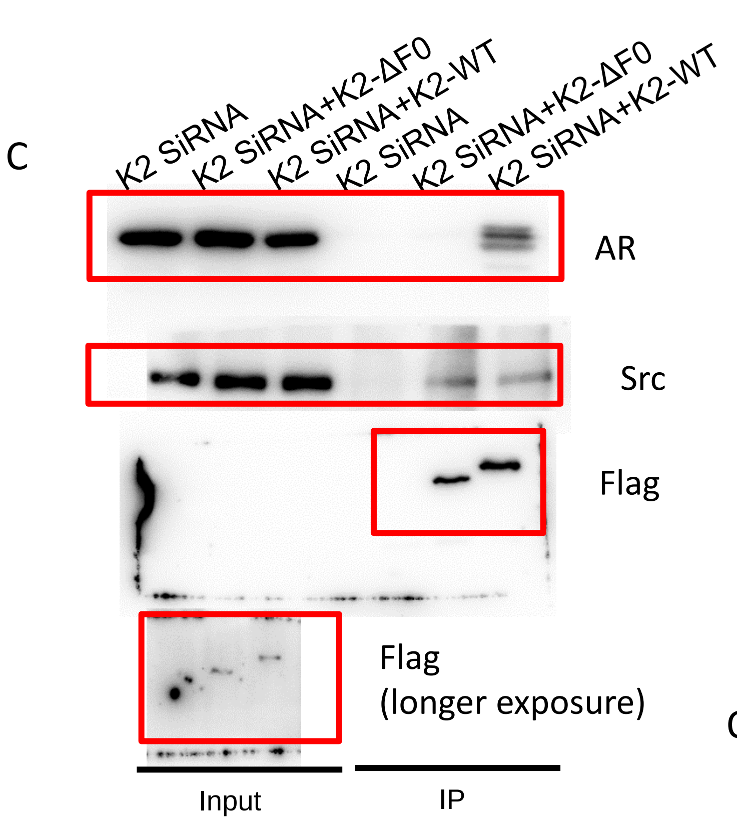

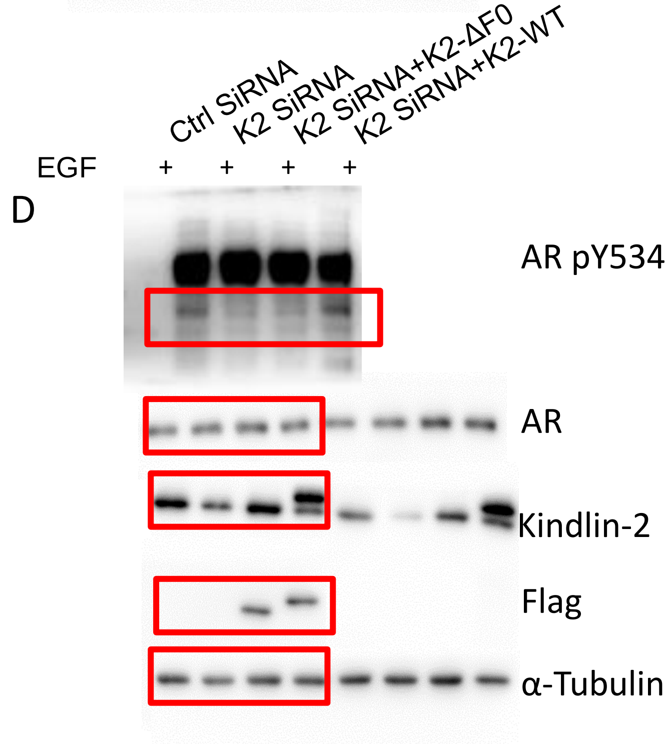

Original western blots for the western blot result in Fig. 3

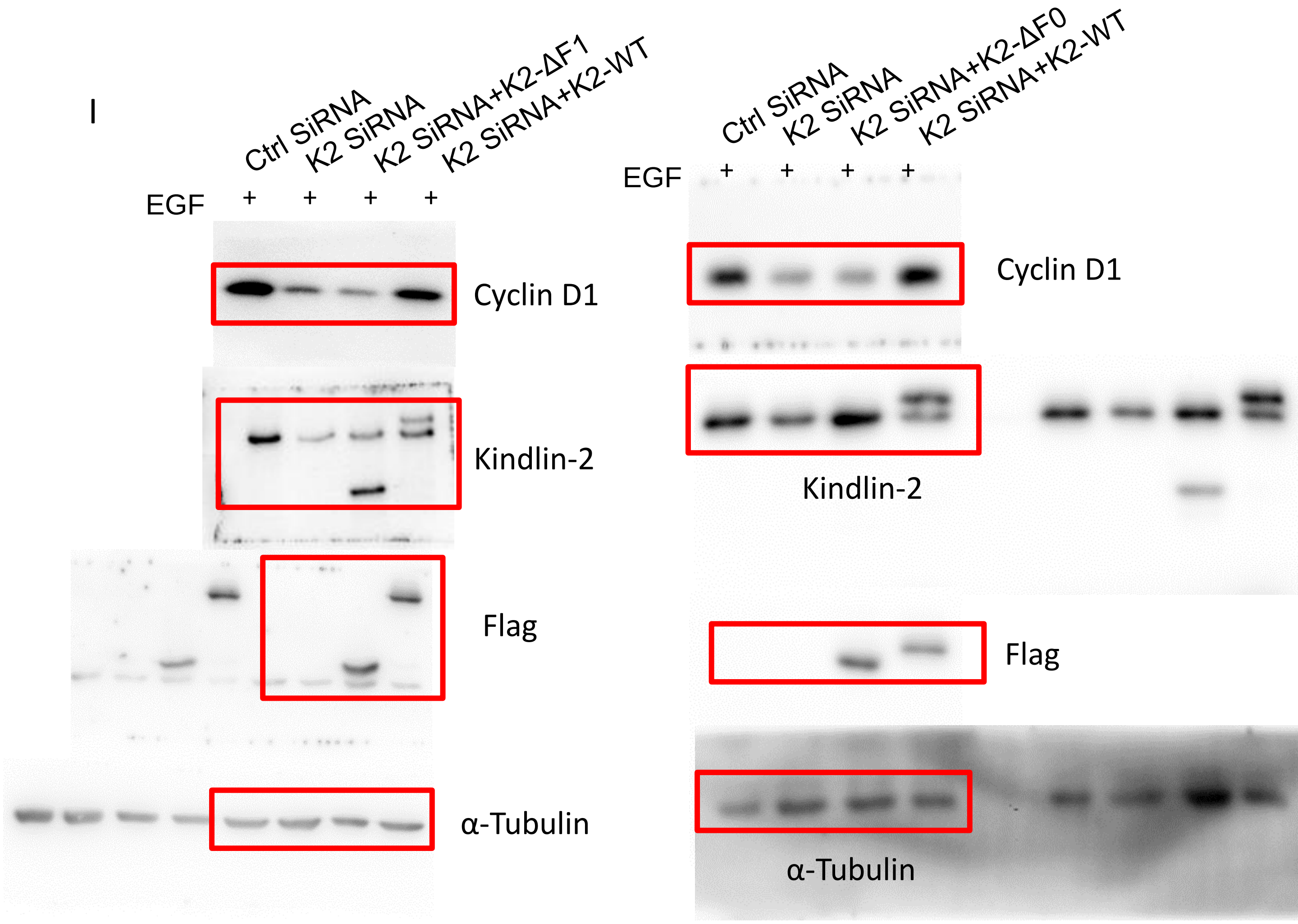

Original western blots for the western blot result in Fig. 3

D

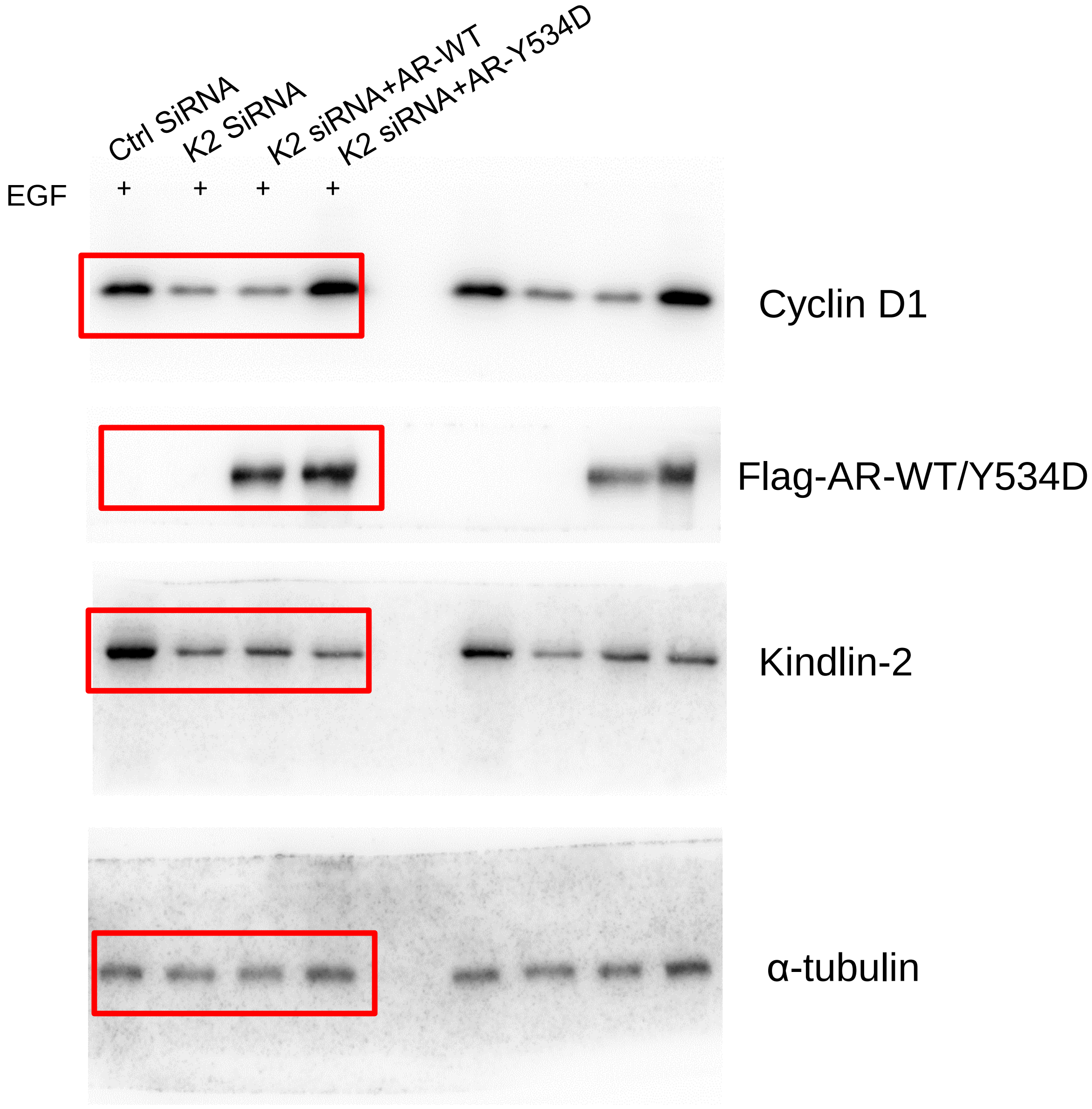

Original western blots for the western blot result in Fig. 4

5C

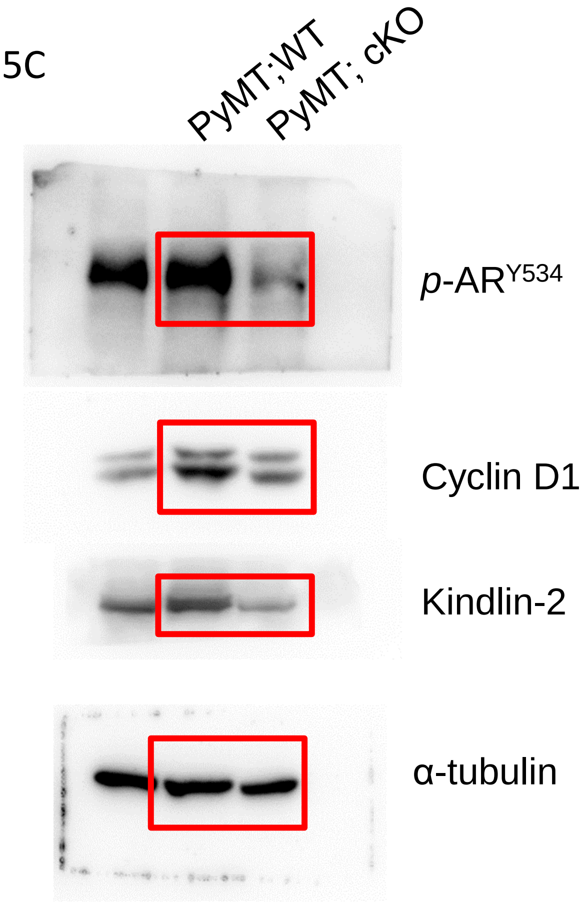

6E

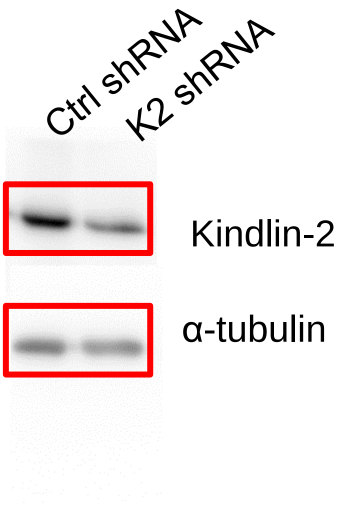

Original western blots for the western blot result in Figs. 5 and 6
